# PRDX1 inhibits ferroptosis by binding to Cullin-3 as a molecular chaperone in colorectal cancer

**DOI:** 10.7150/ijbs.99804

**Published:** 2024-09-23

**Authors:** Yujia Song, Xiaohui Wang, Yuqi Sun, Nianhua Yu, Yajie Tian, Jinli Han, Xianjun Qu, Xinfeng Yu

**Affiliations:** 1 Department of Pharmacology, School of Basic Medical Sciences, Capital Medical University, Beijing, China.; 2 Department of General Surgery, Xuanwu Hospital, Capital Medical University, Beijing, China.

**Keywords:** Peroxiredoxin 1, NRF2, ferroptosis, colorectal cancer

## Abstract

Peroxiredoxin 1 (PRDX1) is a potent antioxidant protein that displays a unique molecular chaperone activity. However, the role of overexpression of PRDX1 in colorectal cancer (CRC) was elusive. Herein, we found that the number of AOM/DSS-induced colitis-associated CRC in PRDX1 knockout mice was significantly lower than that in wild-type mice, concomitant with the downregulation of NRF2 and GPX4. Mechanistically, RNA sequencing results indicated that knockdown of PRDX1 resulted in a significant reduction of NRF2, which further triggered ROS-induced mitochondrial dysfunction and lipid peroxidation-induced ferroptosis in CRC cells. Notably, PRDX1 inhibited NRF2 degradation and promoted NRF2 nuclear translocation, thereby triggering the transcription of GPX4. Immunoprecipitation-mass spectrometry (IP-MS) and Co-immunoprecipitation (Co-IP) assays revealed that PRDX1 could act as a molecular chaperone by binding to CUL3 to inhibit NRF2 ubiquitination. Importantly, the binding of PRDX1 to CUL3 was enhanced by conoidin A but abolished by the PRDX1 Cys83Ser mutant. The inhibitory effects of PRDX1 knockdown on CRC could be attenuated by NRF2 activation or ferrostatin-1 administration *in vivo*. Collectively, these results provide a novel insight into the molecular chaperone activity of PRDX1 in promoting CRC progression through suppression of CUL3-mediated NRF2 degradation, suggesting PRDX1 Cys83 is a potential drug target in inhibiting CRC.

## Introduction

Colorectal cancer (CRC) is the third most common malignancy and the second leading cause of cancer death 1. It has very low rate of early diagnosis, which greatly affects the treatment and prognosis of CRC patients. The imbalance between reactive oxygen species (ROS) generation and the antioxidant defense systems leads to the oxidative stress, which is one of the hallmarks of cancer 2, 3. Oxidative stress can drive malignant transformation through the accumulation of DNA mutations. However, excessive levels of ROS are detrimental to cells, inducing mitochondrial dysfunction, lipid peroxidation and ultimately cell death 4. Thus, various antioxidant systems have evolved to maintain cellular homeostasis.

PRDX1, a member of the typical 2-Cys PRDX subfamily, scavenges intracellular ROS by forming an inter-molecular disulfide bond between the catalytic Cys^52^ and the Cys^173^ residue of two PRDX1 molecules 5. In addition to its antioxidant activity, PRDX1 functions as a molecular chaperone in the form of a high molecular weight (HMW) complex under oxidative stress conditions 5. PRDX1 was previously proposed to be a tumor suppressor gene, supported by the observation of age-dependent hemolytic anemias and/or malignancies in PRDX1 knockout (KO) mice due to increased susceptibility to oxidative DNA damage 6. Accumulating studies have shown that overexpression of PRDX1 exists in numerous types of cancers and enhances their tumorigenic, invasive and metastatic capacities 7, 8. More importantly, PRDX1 has become a therapeutic target and inhibition of PRDX1 in combination with chemotherapy has achieved better anti-tumor efficacy 9, 10. However, the functional role and underlying mechanism of PRDX1 in CRC progression and prognosis remain elusive, which is the focus of our research.

Nuclear factor E2-related factor 2 (NRF2), a leucine zipper transcription factor, plays an important role in maintaining redox homeostasis. In resting cells, NRF2 is sequestered in the cytoplasm with KEAP1, which targets NRF2 for ubiquitination and proteasomal degradation by forming the KEAP1-CUL3-Rbx1 E3 ligase complex. Upon exposure to oxidative stress, NRF2 is stabilized and translocates to the nucleus via the nuclear localization signal (NLS), where it binds to a specific DNA sequence known as the antioxidant response element (ARE), thereby triggering the activation of a cluster of antioxidant and detoxification enzyme genes such as glutathione peroxidase 4 (GPX4), NAD(*P*)H quinone oxidoreductase-1 (NQO-1) and heme oxygenase-1 (HO-1) 11, 12. Recent studies show that NRF2 is a critical mitigator of ferroptosis 13. Ferroptosis, a novel form of non-apoptotic cell death, is characterized by the iron-dependent activation of lipoxygenase and the regulated cell death induced by lipid peroxidation 14. NRF2 has emerged as a key transcription factor in ferroptosis, as RSL-3 and erastin could induce ferroptosis by inhibiting the expression of GPX4 and the cystine/glutamate transporter system SLC7A11/xCT, both of which are downstream targets of NRF2 13. Thus, NRF2-regulated ferroptosis has become a key target in anti-cancer therapies.

In this study, we found that PRDX1 was highly expressed in CRC and associated with poor prognosis of CRC patients. Furthermore, PRDX1 deficiency suppressed azoxymethane (AOM)/dextran sodium sulfate (DSS)-induced colitis-associated CRC in mice possibly by inducing ferroptosis through the NRF2/GPX4 pathway. Notably, knockdown of PRDX1 inhibited NRF2 expression and enhanced ROS-induced mitochondrial dysfunction and lipid peroxidation-induced ferroptosis in CRC cells. Further IP-MS and Co-IP analyses indicated that PRDX1 could interact with cullin-3 (CUL3) as a molecular chaperone, suppress NRF2 ubiquitination-mediated degradation and induce NRF2 nuclear translocation, thereby promoting CRC progression by inhibiting ferroptosis. Importantly, the effect of increased ferroptosis by PRDX1 knockdown on CRC progression could be abrogated by NRF2 activation or ferrostatin-1 (Fer-1) administration *in vivo*.

Taken together, these results provide a novel insight into the molecular chaperone activity of PRDX1 in promoting CRC progression by inhibiting ferroptosis through suppression of CUL3-mediated NRF2 degradation, which will provide a potential drug target for intervention in CRC progression.

## Materials and Methods

### Cell Culture and Cell Transfection

Human CRC cell lines HCT116, SW620, SW480 were purchased from Procell Life Science & Technology Co., Ltd (Wuhan, China). All cells were cultured in RPMI 1640 medium supplemented with 10% fetal bovine serum (FBS) and incubated at 37 °C in a humid atmosphere (5% CO2). Short tandem repeat (STR) analysis was used to authenticate the cells.

To knock down PRDX1 in CRC cells, the cells were transfected with PRDX1 siRNAs (GenePharma, China). The PRDX1 siRNA sequence-1, 2 and 3 were 5′- CAGCCUGUCUGACUACAAATT-3′; 5′-GCACCAUUGCUCAGGAUUATT-3′; 5′-GCCUUCCAGUUCACUGACAAATT -3′. The SW480 and HCT116 cells were infected with lentivirus overexpressing PRDX1 (PRDX1-OE) and their vector control (pHBLV-CMV-MCS-3FLAG-EF1-ZsGreen-T2A-PURO) (Hanbio Biotechnology, Shanghai, China). The HCT116 and SW620 cells were infected with shPRDX1 lentivirus (PRDX1-KD) and the vector control GV248 (GeneChem, Shanghai, China). Puromycin (10 µg/ml) was used to establish stable cell lines. Cells were co-transfected with plasmids of HA-CUL3 and Flag-PRDX1-Mutant (PRDX1-Cys^83^Ser) to overexpress CUL3 and Flag-PRDX1-Mutant by using transfection reagent lipofectamine 2000 (Invitrogen, USA).

### Clinical specimens

We obtained CRC tumor specimens and matched adjacent normal colonic tissues from CRC patients in Xuanwu Hospital of Capital Medical University (Beijing, China). The specimens were surgically removed from patients and confirmed by pathologists (n = 45). Table S1 shows the clinicopathological parameters. Tissues were stored in either RNAlater solution (Qiagen, USA) or 4% Paraformaldehyde Fix Solution for further gene expression analysis. All procedures were approved by the Institutional Review Board of Capital Medical University (Z2023SY009). Informed consents were obtained from the patients and the methodologies were in accordance with the guidelines set by the Declaration of Helsinki.

### RNA sequencing

Total RNA extracted from HCT116 cells transfected with PRDX1 siRNAs (siPRDX1) and negative control (siNC) was subjected to RNA sequencing as previously described 15. The raw data of RNA sequencing have been submitted to the Sequence Read Archive (SRA) under BioProject accession number PRJNA965901.

### Immunohistochemistry (IHC)

IHC was performed on colorectal tissues according to the standard procedures as described previously 15. Sections were stained with antibodies against PRDX1(15816-1-AP), NRF2(16396-1-AP) and GPX4 (67763-1-Ig) (Proteintech, USA) according to the manufacturer's instructions.

### RT-qPCR analysis

RT-qPCR analysis was performed using 7500 Fast Real-Time PCR System (Applied Biosystems, USA) as previously described 15. Relative gene expression was calculated using the 2^-ΔΔCt^ method. All the primer sequences are listed in Table S2.

### Western blotting

Western blotting analysis was performed according to a protocol as described previously 16. Primary antibodies are listed in Table S3. For the detection of PRDX1 dimer and oligomer, the cells were lysed in non-denaturing lysis buffer followed by boiling in non-reducing loading buffer (Applygen, Beijing). Chemiluminescent signals were detected using Super ECL reagent (Millipore) and imaged using the Fluorchem FC3 system (ProteinSimple, USA). β-actin and GAPDH were used as loading controls.

### MTT and colony formation assays

The MTT assay was used to detect tumor cell growth according to the standard protocol (Beyotime, C0009S). Briefly, the cells were seeded in 96-well plates (approximately 4000 cells/well) and incubated for 48 h. Absorbance was measured at a wavelength of 570 nm using a microplate reader.

For the colony formation assay, cells were seeded in 6-well plates (1000 cells/well) and incubated for 14 days. The cells were then fixed with 4% paraformaldehyde (PFA) for 15 min and stained with crystal violet staining solution (Beyotime, C0121) for 20 min at room temperature.

### ROS measurement

ROS in CRC cells were determined by using ROS assay kit (Beyotime, S0033S) according to the manufacturer's instructions. In brief, the cells were seeded in 6-well plates for 24 h, then incubated with 10 µM Dichloro-dihydro-fluorescein diacetate (DCFH-DA) for 20 min. The relative intensity of ROS was immediately evaluated by flow cytometry.

### MitoTracker Red CMXRos staining

To assess mitochondrial morphology and membrane potential, we treated CRC cells with a red-fluorescent dye of MitoTracker Red CMXRos (Beyotime, C1035) according to the manufacturer's instructions. Images of representative cells were acquired using a confocal laser imaging system (TCS SP5, Leica, Germany).

### Malondialdehyde (MDA) measurement

The MDA content of cell samples was determined using the Lipid Peroxidation MDA Assay Kit (Beyotime, S0131S) according to the manufacturer's instructions. Briefly, CRC cells were lysed and reacted with thiobarbituric acid (TBA) to form an MDA-TBA adduct. Absorbance was measured at 532 nm using a spectrophotometer (Molecular Devices, CA, USA).

### Lipid ROS detection

Lipid oxidation was measured using the BODIPY 581/591 C11 assay kit (Thermo Fisher Scientific, MA, USA). Briefly, CRC cells were seeded in 6-well plates and stained with 5 μM C11-BODIPY (581/591) probe. The oxidized C11-BODIPY (O-C11-BODIPY) and reduced C11-BODIPY (R-C11-BODIPY) were observed at excitation/emission wavelengths of 488/510 and 581/591 nm respectively using a laser scanning confocal microscope (TCS SP8, Leica, Germany).

### Immunofluorescence staining

Immunofluorescence staining was performed in CRC cells according to previously described methods 15. Briefly, cells were fixed with -20 °C ethanol for 10min, permeabilized with 0.5% Triton™ X-100 and blocked with 5% BSA. Then the cells were incubated with anti-NRF2 (1:250) (Proteintech, USA) overnight at 4 °C, followed by Alexa Fluor® 594 donkey anti-rabbit secondary antibody (1:1000, Invitrogen, USA) for 1 h at room temperature. The nucleus was stained with DAPI (Beyotime, China).

### Co-immunoprecipitation (Co-IP) assay

Co-IP was performed using the BeaverBeads protein A/G immunoprecipitation kit (BEAVER, China) as previously described 15. Briefly, protein A/G magnetic beads were conjugated with primary antibodies (Table S3) and then incubated with protein lysates overnight at 4 °C to form the immune complexes. The eluted protein was then determined by Western blot analysis.

### Ubiquitination assay

CRC cells were treated with 20 μM MG132 for 6 h and lysed with IP lysis buffer supplemented with protease inhibitor (Roche) and PMSF on ice for 30 min. Ubiquitination of NRF2 was detected by IP with an antibody against NRF2 using protein A/G magnetic beads (BEAVER, China), followed by Western blotting with anti-ubiquitin antibody (Table S3).

### Animal models

PRDX1 knockout (PRDX1-KO) mice were obtained from GemPharmatech Co., Ltd (Jiangsu Province, China). Genotyping was performed by allele-specific PCR analysis using the following primers: Forward-1: 5'-GGCCTCAAACAAGTTATGCAG-3'; Forward-2: 5'-TAAACGATCTTCCCGTTGGCC-3'; Reverse-3: 5'-CGATTAGGTAACTCTGGTTGTC-3'. Mice were maintained under pathogen-free conditions in accordance with NIH guidelines or the Care and Use of Laboratory Animals. All mouse experiments were approved by the Animal Welfare Committee of Capital Medical University. The ethics number was AEEI-2021-194.

The establishment of mouse models of colitis-associated CRC by treatment with AOM/DSS was conducted as previously described 17. In brief, C57BL/6J mice aged at 8 weeks (n = 5 per group) were injected intraperitoneally with AOM (10 mg/kg, Sigma-Aldrich) followed by three cycles of oral administration of 1% DSS (MP Biomedicals) in drinking water. From the start of the second cycle, Fer-1 (10 mg/kg, MedChemExpress, USA) was injected intraperitoneally every other day until the end of the experiment.

Six-week-old female athymic nude mice were purchased from Charles River Laboratories (Beijing, China) and randomly grouped according to body weight (n = 5 for each group). 2× 10^6^ HCT116 cells infected with PRDX1-KD lentivirus (HCT116^PRDX1-KD^) and vector control HCT116^CON-KD^ were injected subcutaneously into nude mice. Mice injected with HCT116^PRDX1-KD^ cells were administered daily with Fer-1 (5 mg/kg), NRF2 agonist Tert-Butylhydroquinone (TBHQ) (10mg/kg) or solvent control. The tumor growth was measured every 3 days using calipers and tumor volumes were calculated using the formula volume (mm^3^) = L × W^2^/2(length L, mm; width W, mm).

### Bioinformatics

TIMER2.0 (http://timer.comp-genomics.org) was used to determine the expression of PRDX1 in various cancers and the correlation of the expression of PRDX1 with NRF2. Gene Expression Profiling Interactive Analysis (GEPIA) (http://GEPIA.cancer-pku.cn/index.html) was used to analyze the correlation of the expression of PRDX1 with GPX4 and overall survival using TCGA datasets in CRC tissues. High and low gene expression levels of the patients were divided by the median cut-off and the log-rank *P* value was presented.

### Statistical analysis

Data are presented as mean ± SD of three independent experiments. One-way analysis of variance (ANOVA) or paired *t*-test was used to analyze the differences between groups. A two-tailed value of *P* < 0.05 was considered statistically significant. GraphPad Prism 8.0 (GraphPad Software Inc., USA) was used for statistical analysis of the data.

## Results

### PRDX1 is upregulated in CRC and promotes tumor progression

**Figure 1:**
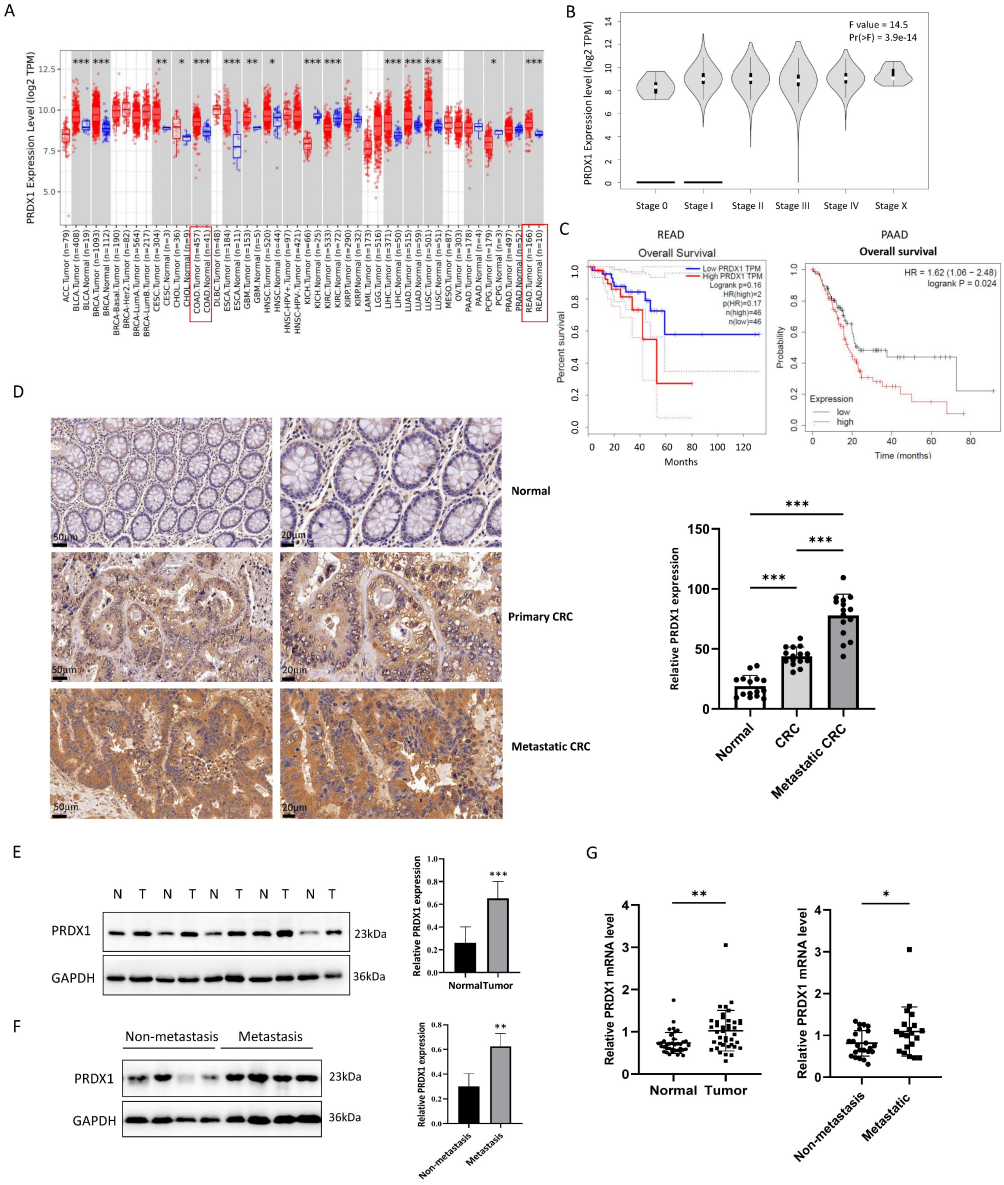
PRDX1 is overexpressed in human CRC tissues and correlated with poor prognosis. (A, B) The relative expression of PRDX1 in different cancers and the correlation with tumor stages of CRC were analyzed by TIMER 2.0 and GEPIA respectively. (C) Overall survival analysis of rectal cancer (READ) and pancreatic cancer (PAAD) patients divided by expression of PRDX1 was analyzed by GEPIA and TIMER 2.0. Patients were divided into high or low expression levels using the median cut-off and the log-rank *P* value was shown. (D) Representative immunohistochemistry images of the expression of PRDX1 in paired normal colonic tissues, primary CRC tissues and metastatic CRC tissues from CRC patients. Relative staining intensity was statistically analyzed (n = 15). Scale bar = 50, 20 μm. ***P < 0.001. (E, F) Western blot analysis of PRDX1 expression in human CRC tissues (T) and adjacent normal tissues (N) and comparison of PRDX1 expression between metastatic and non-metastatic CRC tissues. GAPDH was used as an internal control. **P < 0.01, *** P < 0.001. (G) RT-qPCR analysis of PRDX1 mRNA levels in CRC tissues and adjacent normal tissues and comparison of PRDX1 mRNA levels between non-metastatic (n = 25) and metastatic (n = 20) CRC tissues. *P < 0.05, **P < 0.01.

To assess the expression of PRDX1 in tumor tissues, we analyzed human transcriptomic data available in the TCGA database using TIMER2.0 (http://timer.comp-genomics.org). As a result, the level of PRDX1 mRNA was significantly increased in CRC tissues compared to the normal tissues (Fig.1A). GEPIA (Gene Expression Profiling Interactive Analysis; http://gepia.cancer-pku.cn) showed that the expression of PRDX1 was highly correlated with the progression of tumor stages of CRC (*P* < 0.05) (Fig.1B). Furthermore, the high expression of PRDX1 was associated with poor overall survival in rectal cancer (Logrank *P* = 0.16) although it did not reach statistical significance. However, there was a significant correlation between high expression of PRDX1 and poor overall survival in pancreatic cancer (Logrank *P* = 0.024) (Fig.1C), indicating that PRDX1 is an independent prognostic factor in cancer progression. To further investigate the role of PRDX1 in CRC progression, we performed IHC staining and Western blot assays to assess the expression of PRDX1 in human CRC specimens and adjacent normal tissues. The results indicated that PRDX1 was highly expressed in the cytoplasm of CRC compared with the corresponding adjacent normal tissues (*P* < 0.05) (Fig. 1D, E). Moreover, the level of PRDX1 was much higher in metastatic CRC (n = 20) than in non-metastatic CRC (n = 25) (*P* < 0.05) (Fig. 1F, G). Taken together, these data indicate that PRDX1 overexpression plays an important role in CRC progression and correlates with poor prognosis.

### PRDX1 deficiency suppresses CRC cell growth and AOM/DSS-induced colitis-associated CRC by promoting ferroptosis

Considering the significant clinical relevance of PRDX1 in CRC, we investigated the effect of PRDX1 on tumor cell growth *in vitro*. First, we determined the basal expression levels of PRDX1 in several human CRC cell lines and found that the endogenous expression of PRDX1 was higher in HCT116 and SW620 cells and lower in SW480 cells (Fig. S1A).

**Figure 2:**
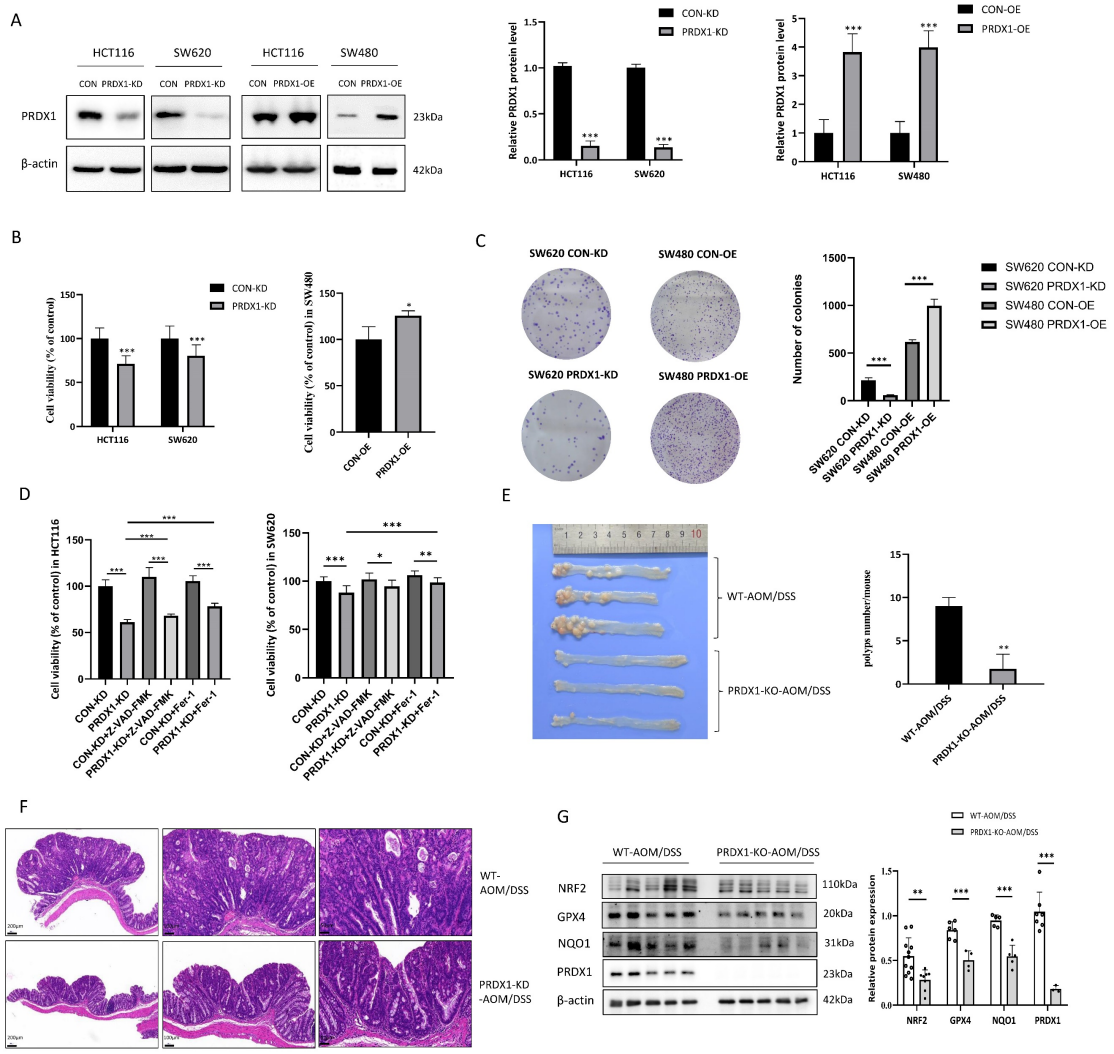
PRDX1 deficiency suppressed CRC cell growth and AOM/DSS-induced inflammatory colorectal adenocarcinoma by enhancing ferroptosis. (A) Western blot analysis of the expression of PRDX1 in CRC cells infected with PRDX1 knockdown (PRDX1-KD) or overexpression (PRDX1-OE) lentivirus. ***P < 0.001. (B, C) MTT and colony formation assays were performed to evaluate the cell proliferation in HCT116^PRDX1-KD^, SW620^PRDX1-KD^ or SW480^PRDX1-OE^ cells compared to controls. (D) MTT assay was performed to evaluate cell viability in SW620^PRDX1-KD^ or HCT116^PRDX1-KD^ cells and their respective control cells treated with or without z-VAD-FMK (20 μM) or Fer-1 (2 μM). (E) Representative images of colons from AOM/DSS-treated WT and PRDX1-KO mice and the number of colonic polyps were statistically analyzed (n = 5). (F) Representative images of colonic tissues from WT and PRDX1-KO mice by HE staining. (G) Western blot analysis of the expression of NRF2, GPX4, NQO-1 and PRDX1 in colonic adenocarcinoma tissues from WT and PRDX1-KO mice as indicated. β-actin was used as an internal control.

Thus, PRDX1 was knocked down in HCT116 and SW620 cells by lentiviral infection to generate the stable cell lines (HCT116^PRDX1-KD^ and SW620^PRDX1-KD^), while PRDX1 was overexpressed in SW480 cells to generate SW480^PRDX1-OE^ stable cells. Since HCT116 cells had a high transfection efficiency, we also generated HCT116^PRDX1-OE^ cells for further functional studies (Fig. 2A). As the results show, knockdown of PRDX1 significantly reduced the cell viability and colony-forming capacity in HCT116 and SW620 cells. In contrast, overexpression of PRDX1 in SW480 cells contributes to cell proliferation (Fig. 2B, C). To investigate whether silencing of PRDX1 leads to a reduction in CRC cell proliferation through increased apoptosis, we analyzed apoptotic proteins in both HCT116^PRDX1-KD^ and SW620^PRDX1-KD^ cells compared to controls. As shown in Fig. S1B, knockdown of PRDX1 substantially enhanced the expression of cleaved PARP, cleaved caspase-9 and Bax in these cells, suggesting that apoptosis contributed to cell death to some extent in PRDX1 knockdown cells compared to controls.

Further analysis of apoptosis by flow cytometry indicated that knockdown of PRDX1 resulted in a small percentage of cell apoptosis which was slightly enhanced by treatment with H_2_O_2_ (Fig. S1C). To further verify whether silencing of PRDX1 induced cancer cell death was also due to ferroptosis, we treated HCT116^PRDX1-KD^ and SW620^PRDX1-KD^ cells with either apoptosis inhibitor (z-VAD) or ferroptosis inhibitor (Fer-1) and then assessed cell viability (Fig. 2D). The results showed that knockdown of PRDX1 significantly reduced cell viability, which was only slightly restored by treatment with z-VAD, but markedly rescued by treatment with Fer-1. These results suggest that ferroptosis may play a predominant role in cell death in PRDX1 knockdown cells compared to controls.

To further explore the potential association of PRDX1 expression with CRC development *in vivo*, we treated PRDX1-KO mice with AOM/DSS to establish the colitis-associated CRC. PCR results showed the genotyping of the PRDX1-KO mice compared with wild-type (WT) mice (Fig. S2A). The results revealed that the number of colonic adenomas in PRDX1-KO mice was significantly lower than that in WT mice (n = 5, *P* < 0.05) (Fig. 2E). Representative images of Hematoxylin-eosin (HE) staining revealed that AOM/DSS-treated WT mice exhibited large adenocarcinomas with disordered crypt structure and glandular lumens, a phenotype that was attenuated in PRDX1-KO mice. The results indicated that PRDX1 deficiency inhibited AOM/DSS-induced colitis-associated CRC (Fig. 2F). Further Western blot analysis revealed that the expression levels of NRF2 and its downstream target genes GPX4 and NQO-1 were reduced in colonic tissues of PRDX1-KO mice compared to those of WT mice (Fig. 2G). This suggests that PRDX1 deficiency suppressed AOM/DSS-induced colitis-associated CRC, possibly by enhancing ferroptosis, as the NRF2/GPX4 signalling pathway played a predominant role in orchestrating ferroptosis.

### Knockdown of PRDX1 triggers ROS-induced mitochondrial dysfunction in CRC cells

**Figure 3:**
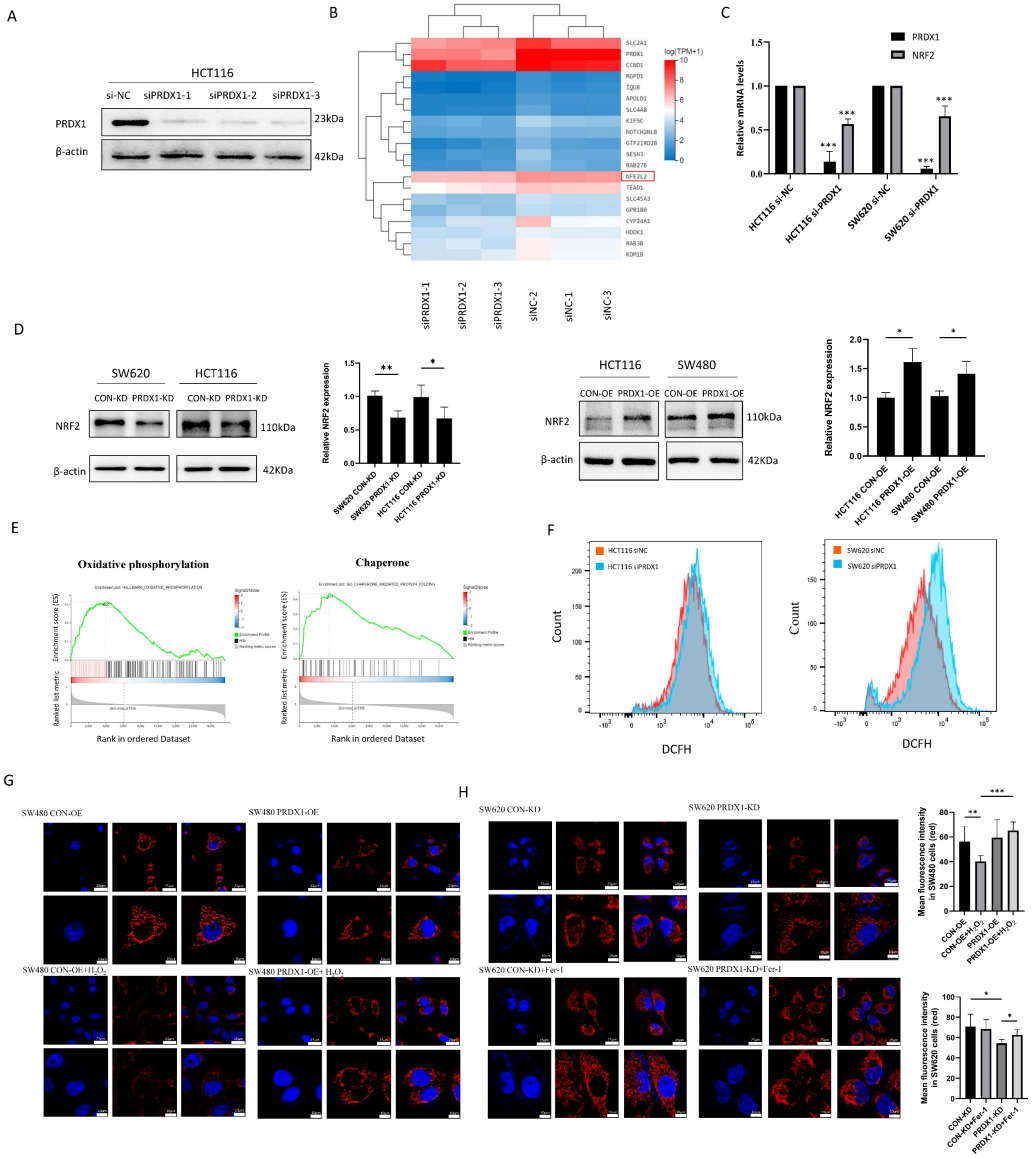
Downregulation of PRDX1 induces mitochondrial dysfunction in colorectal cancer cells. (A) Western blot analysis of the expression of PRDX1 in HCT116 cells transfected with three different PRDX1 siRNA. (B, C) RNA-sequencing was performed in HCT116 siPRDX1 cells and siNC cells. Hierarchical clustering analysis of differentially expressed mRNAs was shown, and downregulation of NRF2 (NFE2L2) was detected by RT-qPCR analysis. (D) Western blot analysis of the expression of NRF2 in SW620^PRDX1-KD^ and HCT116^PRDX1-KD^ or HCT116^PRDX1-OE^ and SW480^PRDX1-OE^ cells compared with controls. (E) GSEA enrichment analysis revealed oxidative phosphorylation and chaperone functions. (F) ROS level was determined by DCFH fluorescence intensity using flow cytometry in HCT116 and SW620 cells transfected with siPRDX1 compared to siNC. (G, H) Mitochondrial membrane potential was measured using MitoTracker Red CMXRos in SW480^CON-OE^ and SW480^PRDX1-OE^ cells treated with or without 100 µM H_2_O_2_ for 0.5 h or in SW620^CON-KD^ and SW620^PRDX1-KD^ cells treated with 2 µM Fer-1 for 24 h as indicated. Statistical analysis of the mean fluorescence intensity was shown. *P < 0.05, **P < 0.01, ***P < 0.001.

To gain a more detailed understanding of genes that might be specifically regulated by PRDX1 in CRC, we performed RNA-Seq analysis and found that there were a total of 44 significantly downregulated genes (Q value < 0.05) in the PRDX1 siRNA group compared to the negative control (Table S4). Western blot analysis was used to prove the downregulation of PRDX1 in HCT116 cells transfected with PRDX1 siRNA (Fig. 3A). The heatmap analysis indicated that NFE2L2 (NRF2) was among the significantly downregulated genes in the PRDX1 siRNA group compared to the control group, which was further validated by RT-qPCR analysis (Fig. 3B, C). Further Western blot analysis suggested the regulatory role of PRDX1 on NRF2 expression by silencing or overexpressing PRDX1 in CRC cells (Fig. 3D). The KEGG analysis showed that the differentially expressed proteins were predominantly enriched in pathways associated with cancer, ubiquitin-mediated proteolysis, and ferroptosis (Fig. S2B). Subsequent gene set enrichment analysis (GSEA) revealed that PRDX1 contributed to CRC development, possibly by regulating oxidative phosphorylation and molecular chaperone functions (Fig. 3E). PRDX1 functions as a critical antioxidant enzyme, facilitating the reduction of peroxides such as hydrogen peroxide. Therefore, it was speculated that PRDX1 may contribute to CRC development by mitigating oxidative stress-related ferroptosis.

To investigate the effects of PRDX1 on oxidative stress, we examined ROS levels in CRC cells transfected with siPRDX1. The knockdown effect of siPRDX1 in SW620 cells was shown in Fig. S2C. The H2DCF fluorescence intensity was significantly enhanced in si-PRDX1 CRC cells, indicating the accumulation of ROS by PRDX1 knockdown (Fig. 3F). The cell-permeant probe MitoTracker Red CMXRos was used to label mitochondria and indicate the loss of mitochondrial membrane potential. Overexpression of PRDX1 in SW480 cells robustly prevented H_2_O_2_-mediated loss of mitochondrial membrane potential. In contrast, knockdown of PRDX1 resulted in the loss of mitochondrial membrane potential, which was significantly suppressed by treatment with Fer-1, a potent and selective ferroptosis inhibitor that prevents damage to membrane lipids (Fig. 3G, H). These results indicate that PRDX1 functions as an antioxidant enzyme and inhibits oxidative stress-induced mitochondrial dysfunction.

### Knockdown of PRDX1 promotes ferroptosis by inhibiting the NRF2/GPX4 pathway in CRC cells

**Figure 4:**
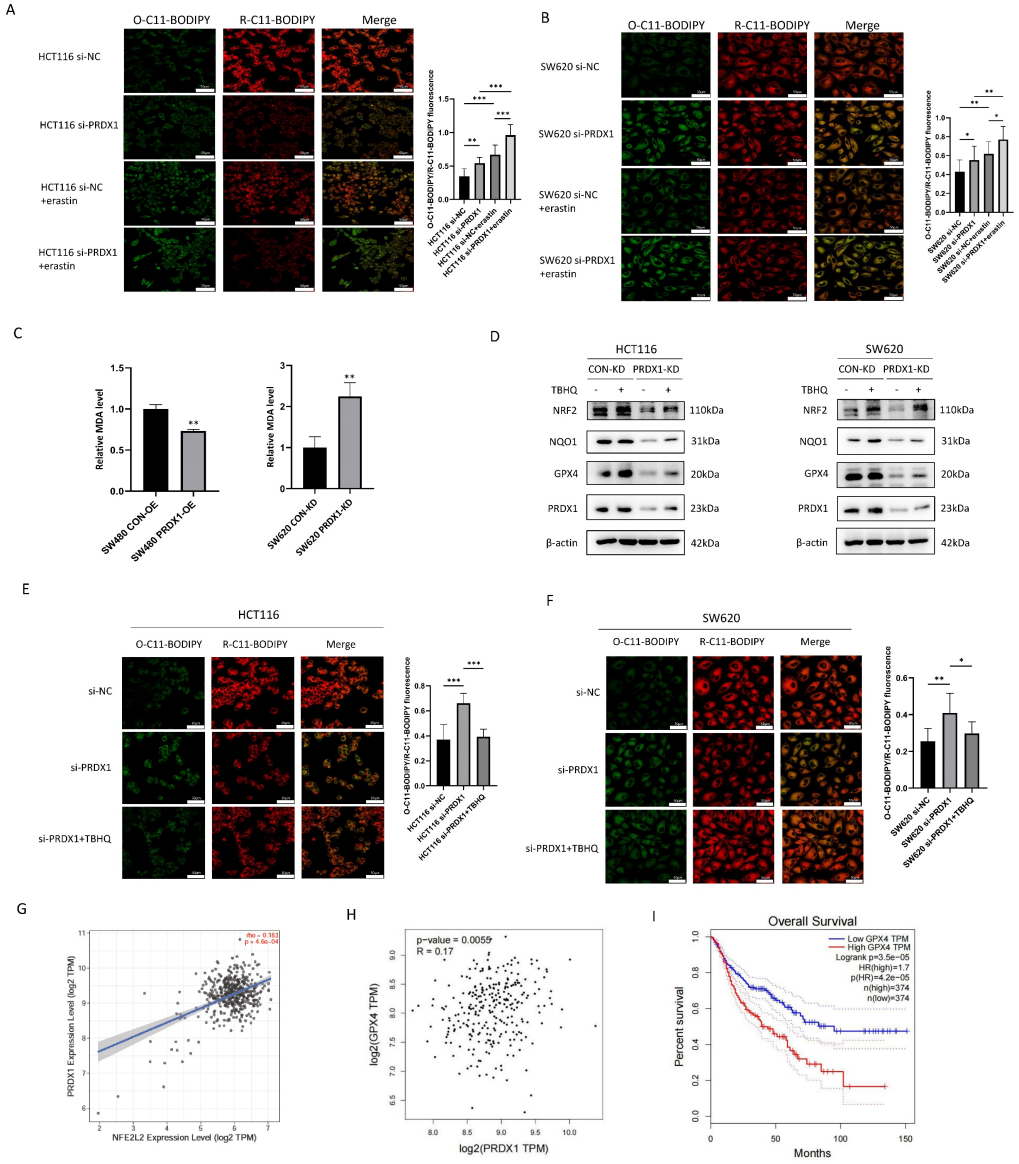
Downregulation of PRDX1 induces ferroptosis through the NRF2/GPX4 pathway in colorectal cancer cells. (A, B) Fluorescence images of BODIPY-C11-stained HCT116 and SW620 cells transfected with PRDX1 siRNA or siNC with or without the treatment with erastin (10 μM). Statistical analysis of the O-C11-BODIPY/R-C11-BODIPY fluorescence ratios between these groups. Blue, DAPI; green, O-C11-BODIPY (oxidized); red, R-C11-BODIPY (reduced). (C) MDA levels were examined in SW480^PRDX1-OE^ or SW620^PRDX1-KD^ cells compared to controls as indicated. (D) Western blot analysis of the expression of NRF2, GPX4, NQO-1 in HCT116^PRDX1-KD^ and SW620^PRDX1-KD^ cells compared to controls in the presence or absence of TBHQ (5 μM) for 24 h. (E, F) Fluorescence images of BODIPY-C11-stained HCT116 and SW620 cells transfected with siNC, PRDX1 siRNA or combined with the treatment with TBHQ (5 μM) for 24 h. Statistical analysis of O-C11-BODIPY/R-C11-BODIPY fluorescence ratios between these groups. *P < 0.05, **P < 0.01, ***P < 0.001. (G, H) Correlation of the expression of PRDX1 with NRF2 and GPX4 was analyzed using TIMER 2.0 and GEPIA. (I) The association of the expression of GPX4 with overall survival in CRC patients was analyzed using GEPIA. Patients were divided into high or low expression levels using the median cut-off and the log-rank *P* value was shown.

Ferroptosis is a novel type of iron-dependent regulatory cell death, characterized by lipid peroxidation and iron accumulation, which has been shown to play an important role in tumor suppression and immunity 18. To further investigate whether knockdown of PRDX1 increases cell ferroptosis, we determined lipid peroxidation by C11-BODIPY581/591 staining. The results showed that knockdown of PRDX1 resulted in increased production of excessive lipid peroxidation, and this increase was strengthened by the addition of canonical ferroptosis inducer erastin (Fig. 4A, B). We further examined the abundance of MDA, the end-product of lipid peroxidation, and found that overexpression of PRDX1 reduced MDA abundance in SW480 cells. Conversely, knockdown of PRDX1 significantly increased MDA levels in SW620 cells (Fig. 4C). Consistently, intracellular Fe^2+^ levels were also determined using the FerroOrange fluorescent probe in HCT116 and SW620 cells transfected with siPRDX1 with or without erastin treatment. The results indicated that knockdown of PRDX1 significantly increased the intracellular Fe^2+^ level, which was further potentiated by treatment with erastin (Fig. S3). Further Western blot analysis confirmed that the expression levels of ferroptosis-related proteins including NRF2, GPX4 and NQO1 were reduced with the downregulation of PRDX1 in CRC cells (Fig. 4D). To further verify whether activation of the NRF2 pathway contributes significantly to the inhibition of ferroptosis, we performed a rescue experiment and found that activation of NRF2 by Tert-Butylhydroquinone (TBHQ) remarkably reversed the downregulation of GPX4 and NQO1 caused by PRDX1 silencing in both HCT116 and SW620 cells (Fig. 4D). Importantly, the increase in lipid peroxidation in CRC cells transfected with si-PRDX1 was largely rescued by treatment with the NRF2 agonist TBHQ, indicating that NRF2 plays a critical role in suppressing ferroptosis induced by PRDX1 knockdown (Fig. 4E, F).

A growing body of evidence indicates that NRF2 is a negative regulator of ferroptosis by inhibiting lipid peroxidation through transcriptional regulation of GPX4 expression 13, 19, 20. Using TIMER 2.0 programme and GEPIA, the expression of PRDX1 was shown to be positively correlated with the expression of NRF2 (NEF2L2) and its target gene GPX4, a major scavenger of phospholipid peroxides in CRC tissues (Fig. 4G, H). Notably, the high expression of GPX4 was associated with poor overall survival in CRC patients (Fig. 4I). Taken together, these results demonstrated that knockdown of PRDX1 promoted ferroptosis by inhibiting the NRF2/GPX4 signalling pathway *in vitro*.

### PRDX1 inhibits NRF2 degradation and promotes NRF2 nuclear translocation by binding to CUL3 as a molecular chaperone

**Figure 5:**
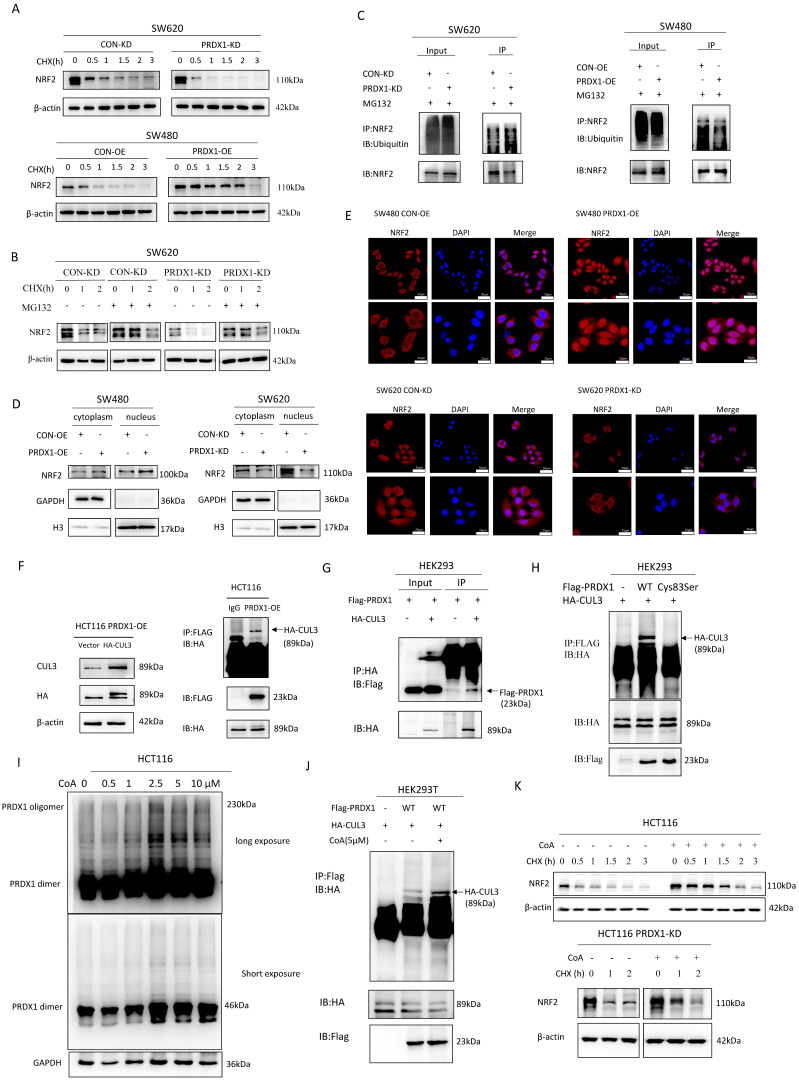
PRDX1 inhibits NRF2 degradation and promotes NRF2 nuclear translocation by binding to cullin-3 as a molecular chaperone to. (A, B) SW620^PRDX1-KD^ cells and SW480^PRDX1-OE^ cells were treated with CHX (100 μg/ml) for different time courses without or with the pretreatment of MG132 (20 μM) for 6 h, the degradation of NRF2 was determined compared with controls. (C) CRC cells were pretreated with MG132 (20 μM) for 6 h, and ubiquitination of PRDX1 was determined in the above cells. (D) Western blot analysis of NRF2 expression in cytoplasmic and nuclear extracts of SW480^PRDX1-OE^ cells and SW620^PRDX1-KD^ cells compared to controls. GAPDH and histone H3 were used as cytoplasmic and nuclear loading controls, respectively. (E) NRF2 localization was evaluated by immunofluorescence in SW480^PRDX1-OE^ or SW620^PRDX1-KD^ cells compared to controls. NRF2 was labelled with Alexa Fluor® 594 donkey anti-rabbit secondary antibody, nuclei were visualized with DAPI, shown in blue. Scale bars = 50, 25 µm respectively. (F) HCT116^PRDX1-OE^ cells were transfected with HA-CUL3 plasmid and vector control, Western blot was conducted to confirm the overexpression of HA-CUL3 and IP with Flag antibody was performed to show the interaction between PRDX1 and CUL3. (G) HEK293T cells were infected with Flag-PRDX1 overexpressing lentivirus, then the cells were transfected with HA-CUL3 plasmid. IP with HA antibody was performed to confirm the interaction between Flag-PRDX1 and HA-CUL3. (H) HEK293T cells were transfected with Flag-PRDX1-WT and Flag-PRDX1-Cys^83^Ser mutant plasmids together with HA-CUL3 for 48 h, cell lysate was pulled down with Flag antibody, the precipitate was immunoblotted with HA antibody as indicated. (I) HCT116 cells were treated with different doses of CoA for 2 h, then Western blot was performed to examine PRDX1 dimer and oligomer by non-denaturing PAGE method. (J) Co-IP was performed to determine the interaction of PRDX1 and CUL3 in HEK293T cells transfected with Flag-PRDX1-WT and HA-CUL3 with the treatment of CoA (5μM) for 2 h. (K) HCT116 or HCT116^PRDX1-KD^ cells were exposed to CHX for different time courses without or with the pretreatment of CoA (5μM) for 2 h, the degradation of NRF2 was determined.

In addition to its potent antioxidant activity, PRDX1 also exerts a unique molecular chaperone activity within the PRDXs family to modulate the stability of key proteins. To determine whether PRDX1 exhibits a molecular chaperone activity in regulating NRF2 stability, we treated CRC cells with cycloheximide (CHX) to determine the turnover rate of NRF2. As shown in Fig. 5A, knockdown of PRDX1 in SW620 cells greatly accelerated the turnover of NRF2 and shortened its half-life. Conversely, overexpression of PRDX1 facilitated the stability of NRF2 and prolonged its half-life. These results revealed that PRDX1 modulates the degradation of NRF2 protein in CRC cells, which could be significantly restored by the proteasome inhibitor MG132 (Fig. 5B). Consistently, silencing of PRDX1 in SW620 cells significantly promoted the ubiquitin conjugation to NRF2, whereas overexpression of PRDX1 in SW480 cells dramatically inhibited NRF2 ubiquitination (Fig. 5C). Taken together, the above data provided compelling evidence that downregulation of PRDX1 promoted ubiquitin-mediated degradation of NRF2 in CRC cells.

Under basal conditions, NRF2 was localized in the cytoplasm and was degraded via the ubiquitin-proteasome pathway 21. Specifically, oxidative stress leads to the translocation of NRF2 to the nucleus to function as an essential transcription factor. To further investigate the potential mechanism by which PRDX1 regulates NRF2, we determined the nuclear-cytoplasmic protein expression of NRF2. Nuclear accumulation of NRF2 was significantly accelerated by PRDX1 overexpression in SW480 cells, which was dramatically attenuated by PRDX1 knockdown in SW620 cells, as assessed by nuclear-cytoplasmic fractionation (Fig. 5D) and immunofluorescence analysis (Fig. 5E). These results revealed a disruption of NRF2-dependent transcriptional programme by suppressing NRF2 nuclear translocation through silencing of PRDX1.

NRF2 is primarily modulated by KEAP1, a substrate adaptor for a CUL3-containing E3 ubiquitin ligase. Thus, the KEAP1-CUL3 E3 ubiquitin ligase complex plays a key role in regulating the ubiquitination and subsequent degradation of NRF2. To identify specific protein interactions with PRDX1 that regulate NRF2 ubiquitination and degradation, we performed immunoprecipitation coupled to mass spectrometry (IP/MS) between HCT116^PRDX1-OE^ and HCT116^CON-OE^ cells. Specifically, a large number of ubiquitin-related proteins were identified (Fig. S4, Table S5). BioGRID (https://thebiogrid.org) was utilized to predict the potential proteins that could interact with PRDX1. As a result, a number of cullins including CUL3 were the candidates (Fig. S4). To further investigate whether PRDX1 could interact with CUL3, we performed Co-IP assays in both HCT116^PRDX1-OE^ and HEK293^PRDX1-OE^ cells transfected with HA-CUL3 plasmid. As a result, Flag-tagged PRDX1 functions as a molecular chaperone that could bind to HA-CUL3 (Fig. 5F, G). The results indicate that PRDX1 specifically interacts with CUL3.

PRDX1 has been shown to contain a Cys^83^ at the putative dimer-dimer interface, which provides the basis for the chaperone activity by forming a doughnut-shaped homodecamer 5. To verify that Cys^83^ is the active site of PRDX1 responsible for molecular chaperone binding to CUL3, we constructed a PRDX1 cysteine mutant (PRDX1-cys^83^Ser). Notably, Co-IP indicated that PRDX1^WT^ interacts specifically with CUL3, whereas the PRDX1-cys^83^Ser mutant was deficient of the chaperone activity and consequently lost the ability to bind to CUL3. These results indicate that Cys^83^ serves as the active site for the chaperone activity of PRDX1, which plays a critical role in binding to CUL3 (Fig. 5H). KEAP1 bridges NRF2 to CUL3, as such, KEAP1-CUL3-E3 ubiquitin ligase complex may be involved in the regulation of NRF2 polyubiquitination and proteasome-mediated degradation. However, the CUL3-mediated NRF2 degradation could be attenuated by PRDX1, a molecular chaperone that binds to CUL3, thereby enhancing NRF2 stability and subsequent nuclear translocation. These findings suggest that PRDX1 may strengthen NRF2 stability by binding to CUL3 through Cys^83^-mediated chaperone activity.

### Increased PRDX1 oligomerization disrupts NRF2 stability by enhancing the binding of CUL3 to PRDX1 oligomers

To investigate whether the oligomerization of PRDX1 potentially interacts with CUL3, we treated CRC cells with Conoidin A (CoA), a PRDX1 inhibitor that binds covalently to the catalytic cysteine. It has been reported that although CoA functions as a major antioxidant by blocking the peroxidative activity of PRDX1 and PRDX2, it could also promote the oligomerization of PRDXs 22. Interestingly, we found that CoA increased the formation of PRDX1 dimer and oligomer in a concentration-dependent manner, particularly the dimers were very prominent in response to 2.5, 5 and 10µM CoA (Fig. 5I). CoA significantly increased the oligomerization of PRDX1 and enhanced the interaction between PRDX1 and CUL3, thereby prolonging the half-life of NRF2 protein in HCT116 cells compared to the control group (Fig. 5J, K). Therefore, the addition of CoA significantly restored the degradation of NRF2 protein in both HCT116 and HCT116^PRDX1-KD^ cells, suggesting that CoA promotes the protein stability of NRF2, possibly due to the increased chaperone activity of PRDX1 oligomers (Fig. 5K). These findings demonstrate that oligomerization of PRDX1 is required for CUL3-mediated NRF2 degradation in CRC cells.

### NRF2 activator or ferroptosis inhibitor promotes CRC progression in PRDX1-KD xenografts *in vivo*

**Figure 6:**
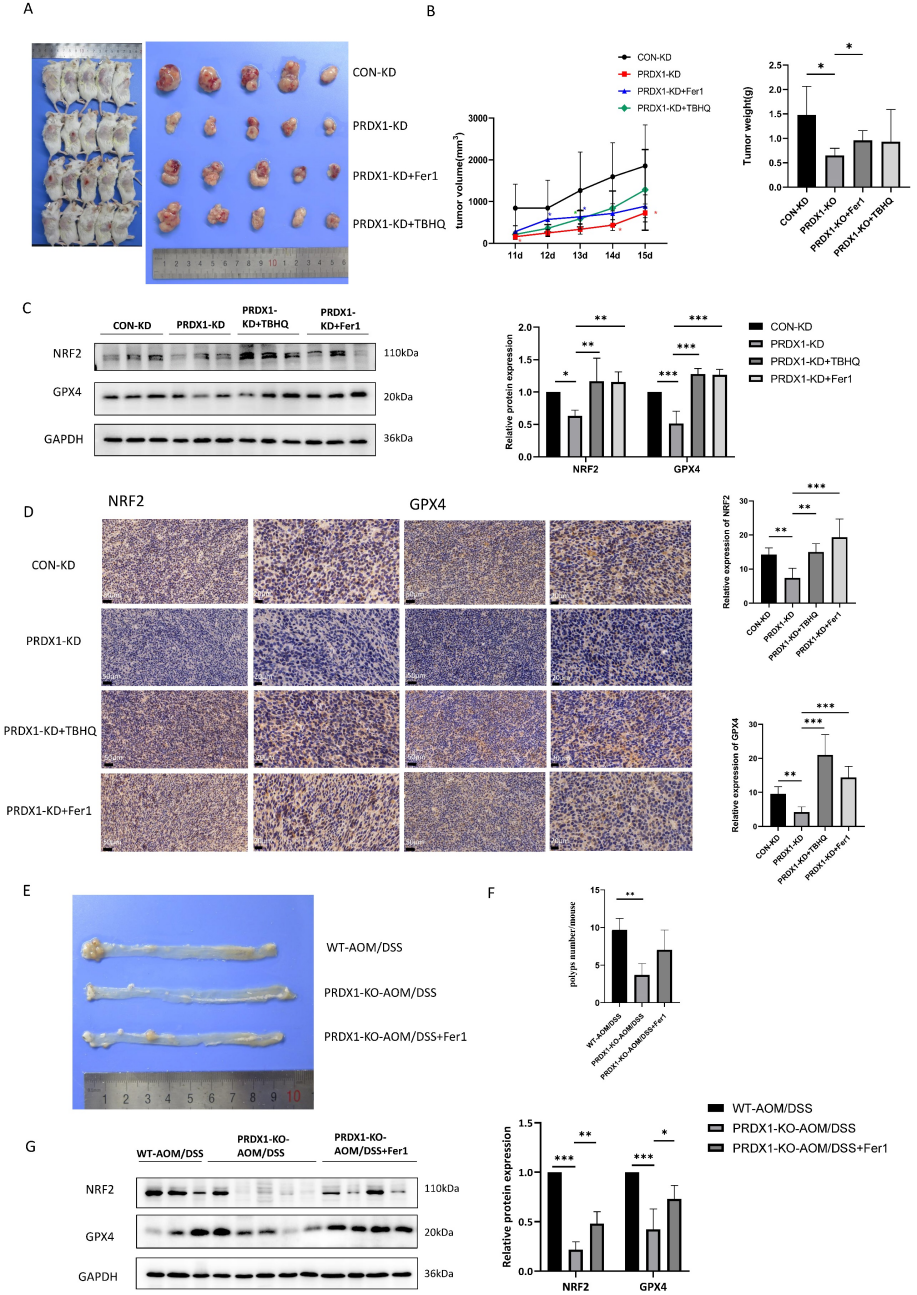
Silencing of PRDX1 inhibits xenograft tumor growth *in vivo* that can be reversed by treatment with Fer-1 and TBHQ. (A) Representative images of subcutaneous tumors transplanted with CT26^CON-KD^ and CT26^PRDX1-KD^ cells in Balb/c mice plus the treatment with Fer-1 or TBHQ as indicated (n = 5). (B) The tumor growth curve and weight of subcutaneous tumors among the groups. (C, D) Expression of NRF2 and GPX4 in subcutaneous tumors was determined by Western blot and IHC staining assays. (E) Representative image of colonic polyps in AOM/DSS-induced WT and PRDX1-KO mice without or with intraperitoneal administration of Fer-1 (10 mg/kg) (n = 5). (F) The number of colonic polyps was analyzed between the groups. (G) Western blot analysis of the expression of NRF2, GPX4 in colonic adenocarcinoma tissues. *P < 0.05, **P < 0.01, ***P < 0.001.

To determine whether downregulation of PRDX1 inhibits the growth of CRC by inducing ferroptosis through suppressing NRF2-mediated transcriptional regulation of antioxidant proteins* in vivo*, we knocked down PRDX1 in murine CRC cell line CT26 and established subcutaneous xenograft tumors using CT26^PRDX1-KD^ cells in the presence or absence of the NRF2 activator TBHQ or ferroptosis inhibitor Fer-1. The results showed that tumor volume and weight were significantly reduced in mice injected with CT26^PRDX1-KD^ cells as compared to the negative control CT26^CON-KD^ group (Fig. 6A, B). Western blot analysis showed that the protein levels of NRF2 and GPX4 in xenograft tumor tissues were significantly reduced by downregulating PRDX1 (Fig. 6C). To verify whether the anti-tumor effects of PRDX1-KD were attributed to downregulation of the NRF2 pathway and enhanced ferroptosis *in vivo*, NRF2 agonist (TBHQ) or Fer-1 treatment were conducted in CT26^PRDX1-KD^ subcutaneous xenograft model. Interestingly, the addition of TBHQ or Fer-1 significantly attenuated the anti-tumor effects of PRDX1 silencing. Importantly, the reduced expression levels of NRF2 and GPX4 in xenograft tumor tissues of the CT26^PRDX1-KD^ group were significantly restored by the treatment with TBHQ or Fer-1 as determined by Western blot and IHC assays (Fig. 6C, D). These findings indicated that downregulation of PRDX1 effectively suppressed tumor growth by enhancing ferroptosis through inhibition of the cytoprotective NRF2-GPX4 signalling pathway *in vivo*.

Consistently, MC38^PRDX1-KD^ and MC38^CON-KD^ stable cell lines were established and subcutaneously injected into nude mice with or without treatment with Fer-1 and TBHQ. These results indicated that knockdown of PRDX1 inhibited xenograft tumor growth, which was rescued by treatment with Fer-1 and TBHQ, probably due to reduced ferroptosis through activating NRF2-GPX4 signalling pathway (Fig. S5).

Supportively, during the process of AOM/DSS-induced colitis-associated CRC, PRDX1-KO mice were also treated with Fer-1. As shown in Fig. 6E, Fer-1 significantly rescued the suppressive effects of PRDX1 knockout on the growth of colonic polyps by inhibiting ferroptosis through activating NRF2-GPX4 signalling pathway (Fig. 6F, G).

### PRDX1 correlates with NRF2 and GPX4 expression in human CRC specimens

**Figure 7:**
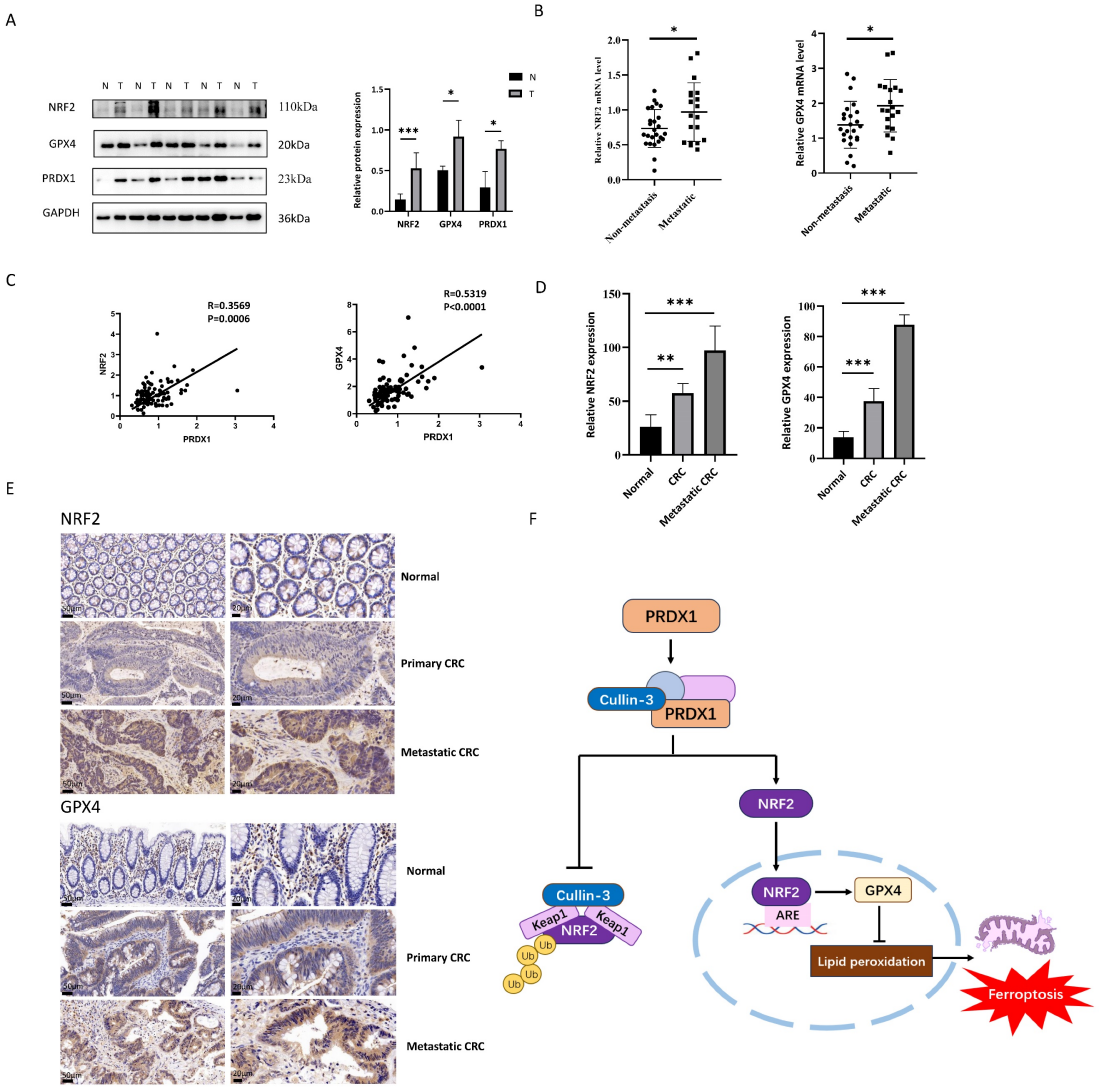
PRDX1 regulates ferroptosis in CRC patients through the NRF2/GPX4 pathway. (A) Western blot analysis of the expression of NRF2, GPX4 and PRDX1 in paired human CRC tissues (T) and adjacent normal colonic tissues (N). (B) RT-qPCR analysis of the mRNA level of NRF2 and GPX4 in metastatic CRC (n = 20) vs. non-metastatic (n = 25) CRC tissues. (C) Spearman statistical analysis was performed to analyze the correlation between PRDX1 and NRF2 or GPX4 mRNA. (D, E) IHC analysis of the expression of NRF2 and GPX4 in CRC tissues (n = 15). Scale bars = 50, 20 µm respectively. (F) Working model depicting the molecular chaperone activity of PRDX1 in suppressing ferroptosis by activating NRF2/GPX4 signalling through binding to cullin-3.

As we have shown in Fig. 1G, PRDX1 was highly expressed in CRC tissues compared to adjacent normal colonic tissues. To further explore the clinical relevance of PRDX1 with ferroptosis in CRC patients, we performed Western blot analysis and found that the expression levels of PRDX1, NRF2 and GPX4 were much higher in CRC tissues than in adjacent normal colonic tissues (Fig. 7A). Notably, the mRNA levels of NRF2 and GPX4 were dramatically higher in metastatic CRC than in non-metastatic counterparts (Fig. 7B). More importantly, there was a significant correlation between the expression of PRDX1 and NRF2 or GPX4 in human CRC tissues (Fig. 7C). Consistently, IHC staining revealed significantly higher protein levels of NRF2 and GPX4 in CRC tissues than in adjacent normal colonic tissues, with even higher levels of NRF2 in metastatic CRC with a predominant nuclear location (Fig. 7D, E). Collectively, these results suggest that PRDX1 contributes to CRC progression by inhibiting ferroptosis, which was negatively dominated by the NRF2/GPX4 signalling pathway in clinical CRC specimens. Our findings provide a new insight into the molecular chaperone activity of PRDX1 in inhibiting ferroptosis by enhancing NRF2 stability through binding to CUL3 (Fig. 7F). Specifically, the binding of PRDX1 to CUL3 prevents the proteasomal degradation of NRF2, induces its nuclear translocation and activates the transcription of several antioxidant proteins such as GPX4 to prevent lipid peroxidation, leading to the inhibition of ferroptosis.

## Discussion

PRDX1 was originally proposed to be a tumor suppressor gene by inhibiting the tyrosine kinase activity of the proto-oncoprotein c-Abl 23. In addition, PRDX1 binds to the transactivation domain of c-Myc and inhibits c-Myc-mediated oncogenic transformation 24. Furthermore, PRDX1 knockout (KO) mice develop age-dependent hemolytic anemias and malignancies 6. These studies suggest that PRDX1 is tumor preventive by acting as H_2_O_2_ scavenging proteins and preventing oxidative stress-induced DNA or protein damage 25. In the present study, we found that PRDX1 was highly expressed in human CRC tissues compared to adjacent normal colonic tissues. In particular, the expression of PRDX1 was higher in metastatic CRC and associated with poor overall survival. Moreover, deficiency of PRDX1 attenuated AOM/DSS-induced colitis-associated CRC. Our findings uncovered a novel role of PRDX1 in promoting CRC progression by inhibiting ferroptosis. Supportively, increasing studies indicated that PRDX1 overexpression promotes tumor growth, chemoresistance and poor prognosis in cancer patients via induction of vascular endothelial growth factor (VEGF) expression 26, activation of c-Jun and transcription factor AP-1 27 or inhibition of cell apoptosis 28. The tumor-promoting effect of PRDX1 in CRC was mainly due to the inhibition of ferroptosis.

Ferroptosis is an iron-dependent, lipid peroxidation-driven cell death cascade that has become a key target in anti-cancer therapies. The transcription factor NRF2 has been proposed to play a central role in the inhibition of ferroptosis, primarily by mitigating oxidative stress and lipid peroxidation 29. NRF2 is activated in response to oxidative stress or toxic chemicals and upregulates the expression of antioxidant enzymes such as NQO1and HO-1 30. Recently, accumulating evidence suggests that NRF2 exerts anti-ferroptotic effects by regulating iron homeostasis and lipid metabolism 31. Lipid peroxides are formed by oxidizing polyunsaturated fatty acids (PUFAs) in the cell membrane or organellar membranes, resulting in the end-products of 4-hydroxynenal (4-HNE) and MDA. NRF2 controls the expression of several target genes including GPX4 and SLC7A11 and plays a key role in reducing lipid peroxides, maintaining membrane integrity and suppressing the ferroptosis cascade 13.

In this study, we found that knockdown of PRDX1 augmented cell ferroptosis as evidenced by increased MDA levels and lipid peroxidation. PRDX1 exerted its anti-ferroptosis effects by regulating the expression of NRF2 and its target genes. Importantly, we uncovered a novel role of PRDX1 in suppressing ferroptosis by regulating NRF2 ubiquitination and degradation. PRDX1 has previously been shown to be a target gene of NRF2 32, while our results indicate that PRDX1 reciprocally regulates the protein stability of NRF2, suggesting a potential positive feedback loop between NRF2 and PRDX1 expression.

NRF2 is regulated at the protein level by several ubiquitin ligases 33. In the canonical pathway, NRF2 is fine-tuned by KEAP1, an adaptor for the CUL3-based ubiquitin E3 ligase and targeted for proteasomal degradation. In addition to the well-established KEAP1-CUL3-dependent pathway that modulates NRF2 in the cytoplasm, a KEAP1-independent mechanism involving β-TrCP (β-transducin repeat-containing protein) also operates in the nucleus 34. The β-TrCP-Cullin1(CUL1) complex has been identified as an alternative pathway for NRF2 degradation. Generally, in the absence of oxidative stress, NRF2 is efficiently ubiquitinated by the KEAP1-CUL3 E3 ligase. Under oxidative stress, the degradation of NRF2 is inhibited and newly synthesized NRF2 translocates to the nucleus and plays an important role in mitochondrial function, metabolic reprogramming and cell survival 21, 35. In this study, CHX chase assay indicated that knockdown of PRDX1 induced the turnover of NRF2 protein and shortened its half-life. To further investigate the mechanism of PRDX1 in regulating NRF2 stability, we performed IP-MS and Co-IP and found that PRDX1 could bind to CUL3 via its molecular chaperone activity, forming a CUL3-KEAP1 ubiquitin complex.

It has been reported that the hyperoxidized form of PRDX1 enhanced the chaperone activity with a redox-dependent conformational change, converting from low-molecular-weight (LMW) to HMW complexes 36. PRDX1 possesses a unique chaperone activity and a cysteine (Cys^83^) at the putative dimer-dimer interface might influence the oligomeric structure and consequently the functions of PRDX1 5. peroxiredoxin homodimers may assemble to form decamers, which depend on many factors such as redox state, pH, and post-translational modifications. It is widely recognized that PRDX1 exerts the molecular chaperone activity in the form of oligomers, which is dramatically enhanced under oxidative stress conditions 10, 37. oligomeric PRDX1 directly associates with p53 or transcription factors such as c-Myc, NF-κB and androgen receptor (AR), thereby modulating their bioactivities 24, 38, 39. Additionally, PRDX1 in the cytoplasm has anti-apoptotic potential through direct or indirect interactions with several ROS-dependent effectors, including ASK1, GSTpi/JNK and c-Abl kinase 7. In addition to its peroxidase activity, PRDX1 also plays an important role in regulating cellular signalling in proliferation and apoptosis by acting as a molecular chaperone. Recently, accumulating evidence suggests that PRDX1 negatively regulates TLR4 signalling for NF-κB activation by inhibiting TRAF6 ubiquitin-ligase activity 40. PRDX1 oligomerization promotes cullin-5 neddylation-mediated NOXA degradation and contributes to etoposide resistance in CRC 41. Our results indicate that PRDX1 exhibits the antioxidant activity and protects against mitochondrial dysfunction by scavenging ROS. Under oxidative stress, PRDX1 is hyperoxidized to form oligomers and interacts with CUL3 to inhibit CUL3-KEAP1 mediated degradation of NRF2. Interestingly, CoA, as an inhibitor of antioxidant activity of PRDX1 and PRDX2, has also been shown to crosslink catalytic cysteines of PRDX1 in a stable oxidized decamer 42, which could influence the molecular chaperone activity of PRDX1 by forming HMW complexes. Further mechanism needs to be elucidated. Importantly, it has been reported that Cys^83^ plays a critical role in regulating the chaperone activity of PRDX1 by maintaining the oligomeric structure 5. We found that mutation of Cys^83^ of PRDX1 abolished the interaction between PRDX1 and CUL3, suggesting that cysteine 83 of PRDX1 plays an important role in modulating the ubiquitination and degradation of NRF2.

Taken together, these results revealed a novel role of PRDX1 in triggering CRC progression by inhibiting ferroptosis through enhancing NRF2 stability by binding to CUL3, highlighting a pivotal molecular chaperone activity of PRDX1. It has been proposed that the active site cysteine 83 of PRDX1 may be a potential drug target for intervening CRC progression.

## Supplementary Material

Supplementary figures and tables.
