# Supplementary material for: PRDX1 inhibits ferroptosis by binding to Cullin-3 as a molecular chaperone in colorectal cancer
Source: Int J Biol Sci. 2024 Sep 23;20(13):5070–86. doi: 10.7150/ijbs.99804 (PMC11489176; doi:10.7150/ijbs.99804)
Supplement: Supplementary file 1 — Supplementary figures and tables. [file ijbsv20p5070s1.pdf]

Fig. S1

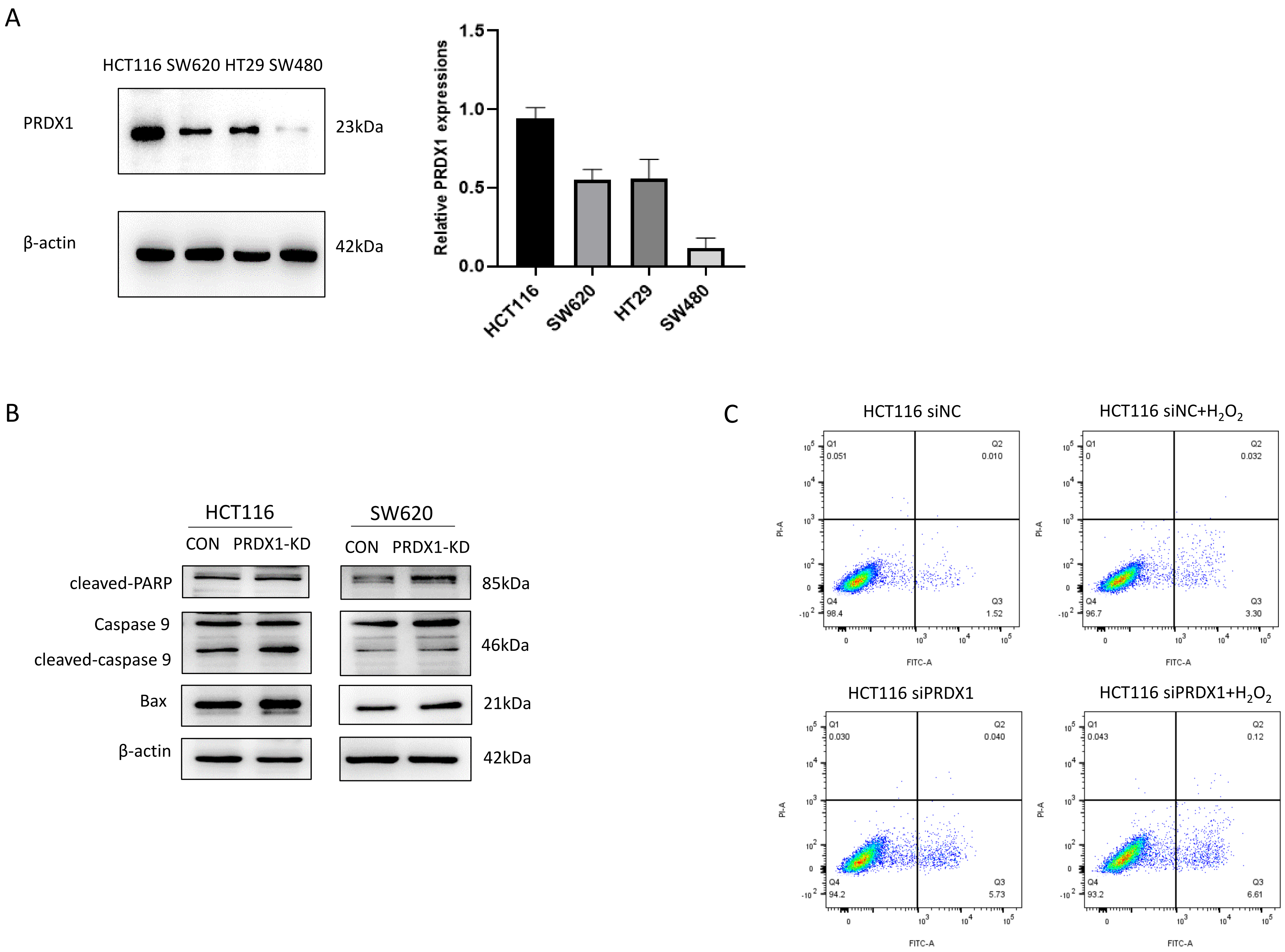
A
HCT116 SW620 HT29 SW480
PRDX1
23kDa
β-actin
42kDa
Relative PRDX1 expressions
1.5
1.0
0.5
0.0
HCT116
SW620
HT29
SW480
B
HCT116
SW620
CON
PRDX1-KD
cleaved-PARP
Caspase 9
cleaved-caspase 9
Bax
β-actin
85kDa
46kDa
21kDa
42kDa
C
HCT116 siNC
HCT116 siNC+H2O2
HCT116 siPRDX1
HCT116 siPRDX1+H2O2
Q1 0.051
Q2 0.010
Q3 1.52
Q4 98.4
Q1 0
Q2 0.032
Q3 3.30
Q4 96.7
Q1 0.030
Q2 0.040
Q3 5.73
Q4 94.2
Q1 0.043
Q2 0.12
Q3 6.61
Q4 93.2
PI-A
FITC-A

Fig. S2

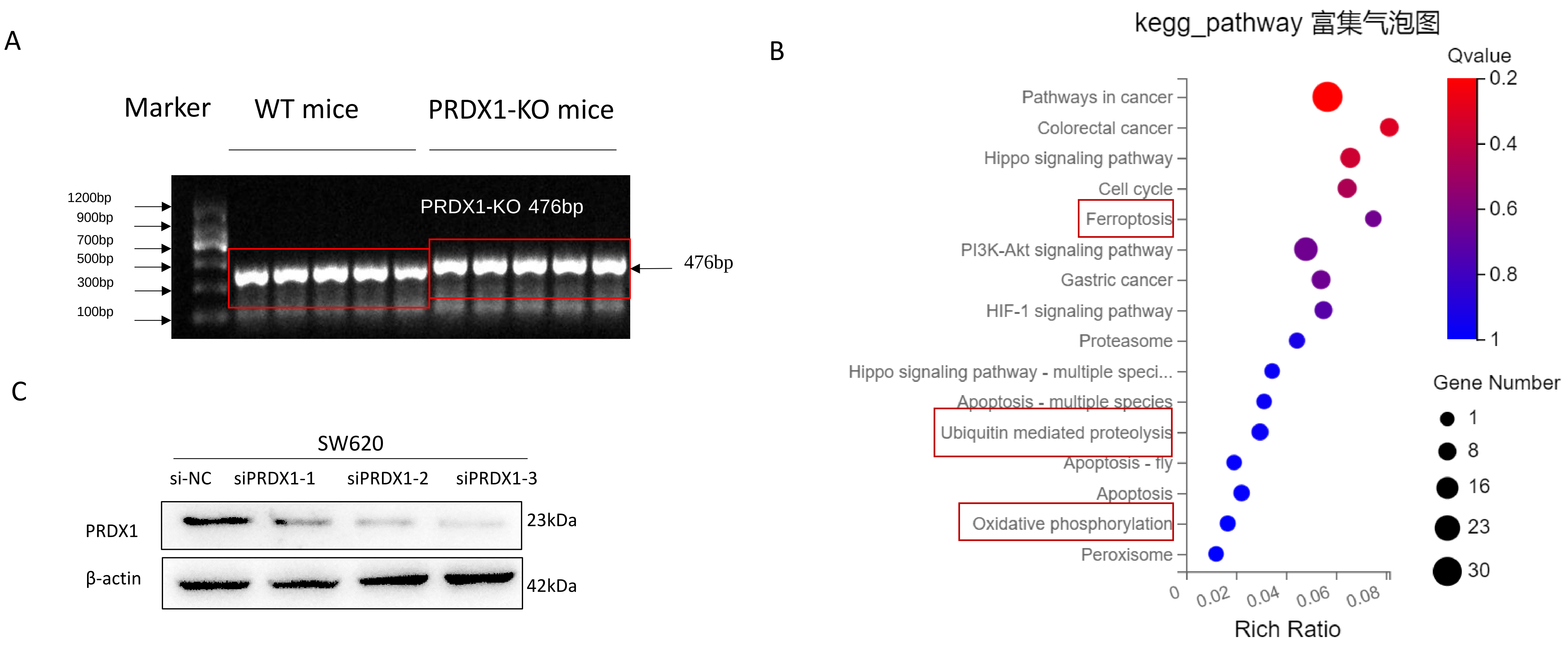
A
Marker
WT mice
PRDX1-KO mice
1200bp
900bp
700bp
500bp
300bp
100bp
PRDX1-KO 476bp
476bp
B
kegg_pathway 富集气泡图
Pathways in cancer
Colorectal cancer
Hippo signaling pathway
Cell cycle
Ferroptosis
PI3K-Akt signaling pathway
Gastric cancer
HIF-1 signaling pathway
Proteasome
Hippo signaling pathway - multiple speci...
Apoptosis - multiple species
Ubiquitin mediated proteolysis
Apoptosis - fly
Apoptosis
Oxidative phosphorylation
Peroxisome
0
0.02
0.04
0.06
0.08
Rich Ratio
Qvalue
0.2
0.4
0.6
0.8
1
Gene Number
1
8
16
23
30
C
SW620
si-NC
siPRDX1-1
siPRDX1-2
siPRDX1-3
PRDX1
β-actin
23kDa
42kDa

Fig. S3

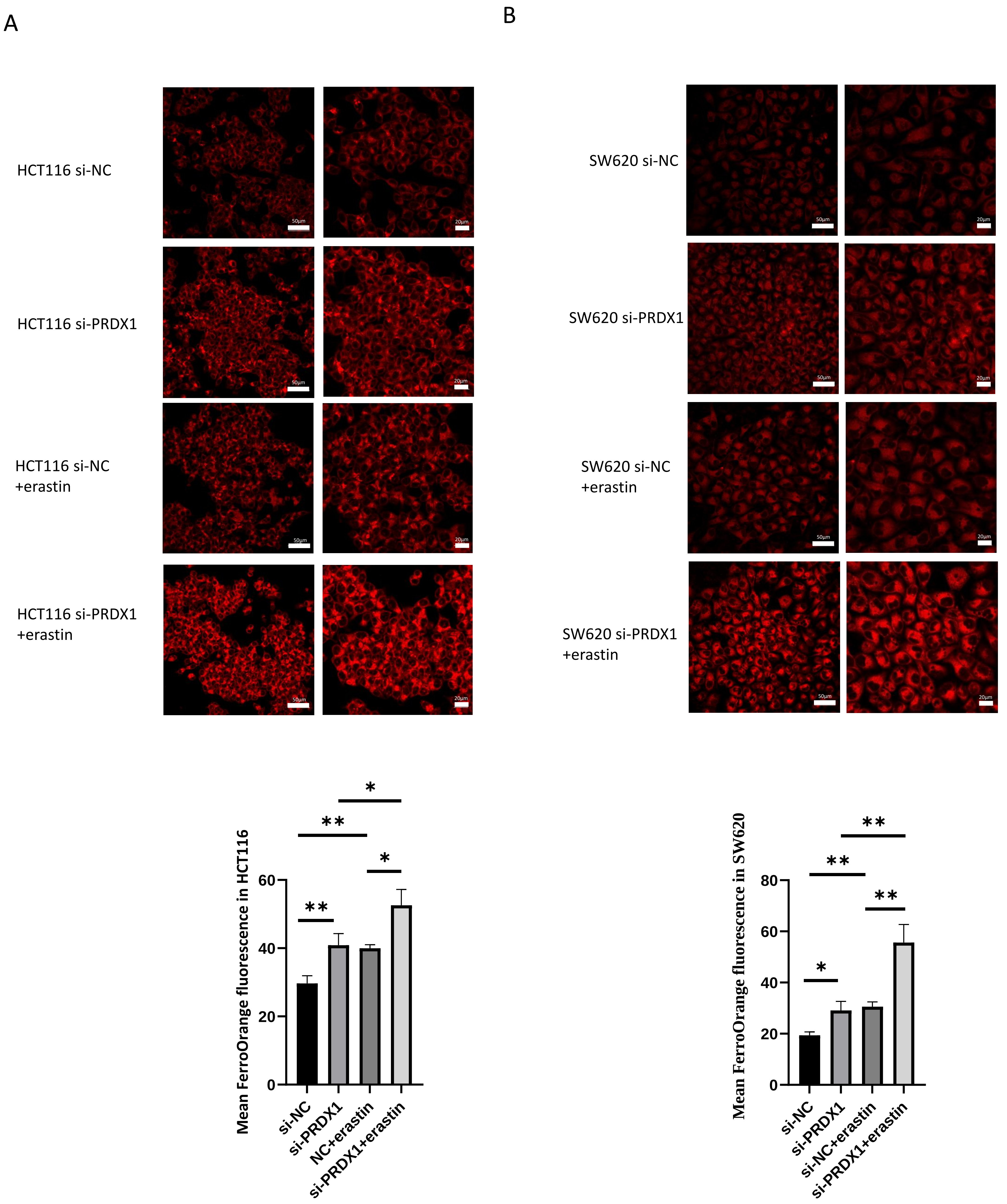

A
B
HCT116 si-NC
HCT116 si-PRDX1
HCT116 si-NC
+erastin
HCT116 si-PRDX1
+erastin
SW620 si-NC
SW620 si-PRDX1
SW620 si-NC
+erastin
SW620 si-PRDX1
+erastin
Mean FerroOrange fluorescence in HCT116
0
20
40
60
si-NC
si-PRDX1
NC+erastin
si-PRDX1+erastin
Mean FerroOrange fluorescence in SW620
0
20
40
60
80
si-NC
si-PRDX1
si-NC+erastin
si-PRDX1+erastin

Fig. S4

A

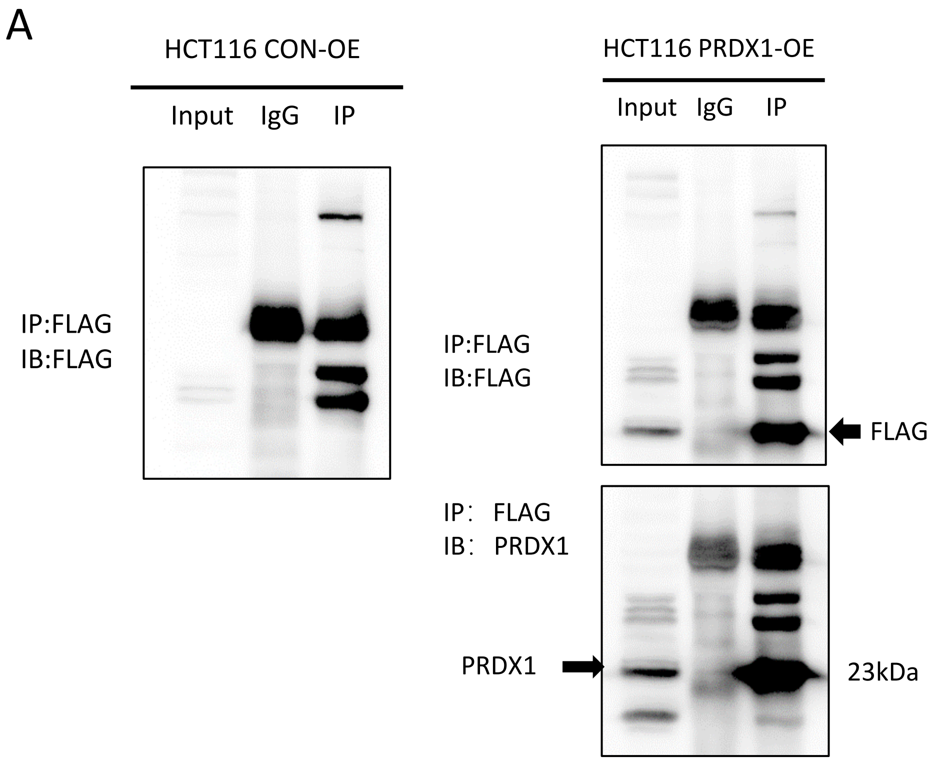
HCT116 CON-OE
Input IgG IP
IP:FLAG
IB:FLAG
HCT116 PRDX1-OE
Input IgG IP
IP:FLAG
IB:FLAG
FLAG
IP: FLAG
IB: PRDX1
PRDX1
23kDa

B

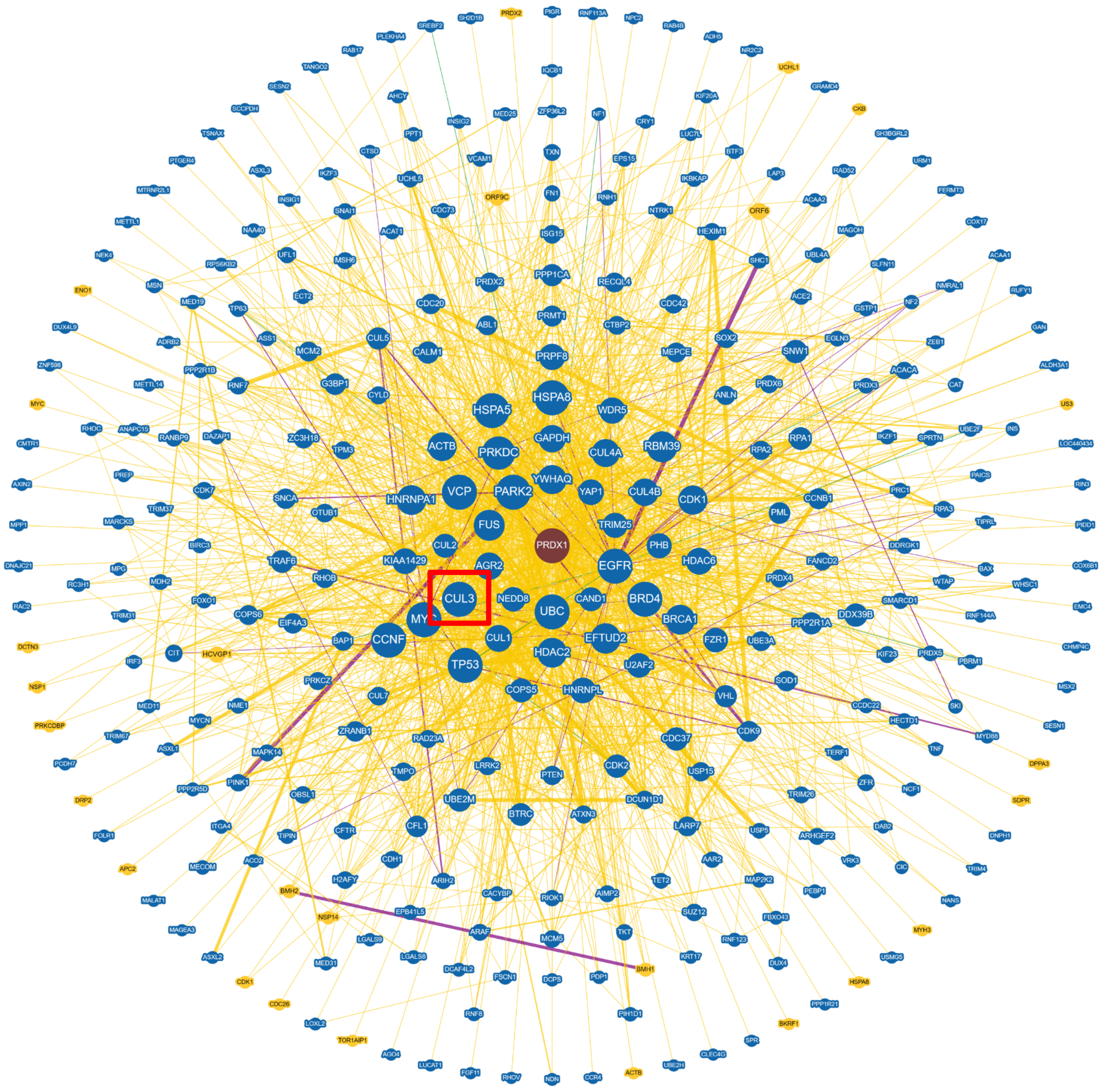
HSPA5
HSPA8
GAPDH
PRKDC
ACTB
WDR5
CUL4A
RBM39
VCP
PARK2
YWHAQ
YAP1
CUL4B
CDK1
HNRNPA1
FUS
TRIM25
PRDX1
PHB
HDAC6
CUL2
KIAA1429
AGR2
EGFR
CUL3
NEDD8
UBC
CAND1
BRD4
BRCA1
CCNF
CUL1
HDAC2
EFTUD2
TP53
COPS5
HNRNPL

Fig. S5

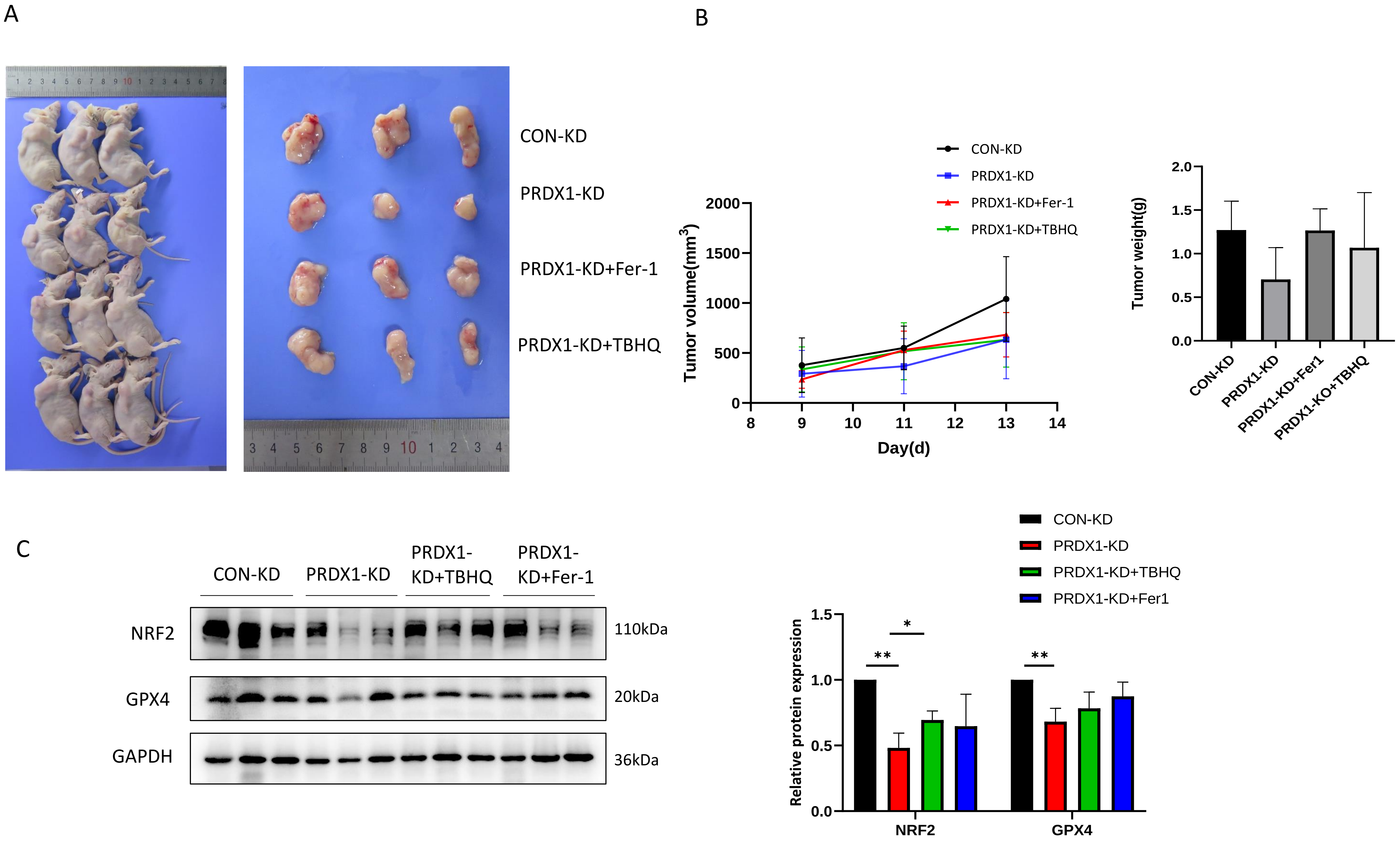

A
CON-KD
PRDX1-KD
PRDX1-KD+Fer-1
PRDX1-KD+TBHQ
B
CON-KD
PRDX1-KD
PRDX1-KD+Fer-1
PRDX1-KD+TBHQ
Tumor volume(mm3)
Day(d)
Tumor weight(g)
CON-KD
PRDX1-KD
PRDX1-KD+Fer1
PRDX1-KO+TBHQ
C
CON-KD
PRDX1-KD
PRDX1-KD+TBHQ
PRDX1-KD+Fer-1
NRF2
GPX4
GAPDH
110kDa
20kDa
36kDa
CON-KD
PRDX1-KD
PRDX1-KD+TBHQ
PRDX1-KD+Fer1
Relative protein expression
NRF2
GPX4

Fig. S1 (A) Western blot analysis of the expression of PRDX1 in HCT116, SW620, HT29 and SW480 cells. β-actin was used as an internal control. (B) Western blot analysis of the expression of cleaved-PARP, cleaved caspase 9, Bax expression in HCT116$^{PRDX1\text{-}KD}$ and SW620$^{PRDX1\text{-}KD}$ cells compared to controls. β-actin was used as an internal control. (C) Cell apoptosis was determined using Annexin V-FITC and PI staining by flow cytometry analysis.

Fig. S2 (A) Genotyping of the PRDX1-KO mice by allele-specific PCR analysis. The target band is 476bp. Genotyping of WT mice was used as a control. (B) KEGG pathway analysis of the differentially expressed genes between HCT116 siPRDX1 and siNC groups. The main pathways were highlighted in red. (C) Western blot analysis of the expression of PRDX1 in SW620 cells transfected with three different PRDX1 siRNA.

Fig. S3 The FerroOrange fluorescent probe (1 μM) was used to determine intracellular Fe^{2+} levels in HCT116 or SW620 cells transfected with siPRDX1 and siNC with or without the treatment with erastin (10 μM). Statistical results of mean FerroOrange fluorescence intensity were indicated.

Fig. S4 (A) HCT116$^{CON\text{-}OE}$ and HCT116$^{PRDX1\text{-}OE}$ cells were subjected to IP with anti-FLAG antibody and Western blot analysis of FLAG-PRDX1 expression in HCT116$^{PRDX1\text{-}OE}$ cells before Mass-Spectrometry. (B) The interaction between PRDX1 and CUL3 was predicted by BioGRID

(https://thebiogrid.org).

Fig. S5 (A) Representative images of subcutaneous tumors transplanted with MC38^{CON-KD} and MC38$^{PRDX1-KD}$ cells in nude mice plus the treatment with Fer-1 or TBHQ as indicated (n = 3). (B) The tumor growth curve and weight of subcutaneous tumors among the groups. (C) The expression levels of NRF2 and GPX4 in subcutaneous tumors were determined by Western blot analysis.

Table S1. Demographic information of colorectal cancer patients

	Variables	Number	Percentage (%)
Age	<=70	27	60.0
	>70	18	40.0
Gender	Male	26	57.8
	Female	19	42.8
Location	Colon	34	75.6
	Rectum	11	24.4
Depth of invasion	T_{1-2}	11	24.4
	T_{3-4}	34	75.6
Lymph node metastasis	N_0	21	46.7
	N_{1-2}	24	53.3
Distant metastasis	M_0	25	55.6
	M_1	20	44.4

Table S2. The sequences of primers for RT-qPCR

Gene	Primer Sequence (5′ to 3′)	Species
PRDX1	F: CAACTGCCAAGTGATTGGTG<br>R: TGATCTGCCGAAGAATACCC	*Homo sapiens*
NRF2	F: GCGACGGAAAGAGTATGAGC<br>R: TGGGAGTAGTTGGCAGATCC	*Homo sapiens*
GPX4	F: TCAGCAAGATCTGCGTGAAC<br>R: GGGGCAGGTCCTTCTCTATC	*Homo sapiens*

Table S3. The antibodies and application

Antibodies	Company	Application
β-actin (66009-1-Ig)	Proteintech	WB
PRDX1 (15816-1-AP)	Proteintech	WB/IHC
NRF2 (16396-1-AP)	Proteintech	WB/IHC/IF
GPX4 (67763-1-Ig)	Proteintech	WB/IHC
NQO1 (A180)	Santa Cruz	WB
PARP (L080950)	PTM Bio	WB
Caspase 9 (10380-1-AP)	Proteintech	WB
Bax (50599-2-Ig)	Proteintech	WB
Ubiquitin (L020707)	PTM Bio	Co-IP/WB
FLAG tag (20543-1-AP)	Proteintech	Co-IP/WB
GAPDH (60004-1-Ig)	Proteintech	WB
HA tag (51064-2-AP)	Proteintech	Co-IP/WB
Histone H3 (4499)	Cell Signaling Technology	WB
Normal Rabbit IgG (2729)	Cell Signaling Technology	Co-IP

Table S4. The downregulated genes in HCT116 siPRDX1 normalized to siNC

Gene ID	Gene Symbol	log2 (siPrdx1/siNC)	Q value (siPrdx1/siNC)	P value (siPrdx1/siNC)
5052	'PRDX1'	-3.408226577	1.18E-57	6.68E-62
154865	'IQUB'	-2.573033624	0.04213658	3.89E-04
400966	'RGPD1'	-1.675634422	0.029279443	2.34E-04
1591	'CYP24A1'	-1.59564471	0.00207419	6.00E-06
9498	'SLC4A8'	-1.514491282	0.026866287	1.99E-04
389524	'GTF2IRD2B'	-1.470700195	0.040453887	3.67E-04
143686	'SESN3'	-1.401628064	0.012288167	6.97E-05
81575	'APOLD1'	-1.300341423	0.045366703	4.35E-04
5874	'RAB27B'	-1.173962631	0.008178988	4.13E-05
3800	'KIF5C'	-1.084905562	0.003477128	1.16E-05
6513	'SLC2A1'	-1.064049212	0.004750001	1.86E-05
595	'CCND1'	-1.0639592	0.024556043	1.70E-04
22989	'MYH15'	-0.979167931	1.51E-04	2.06E-07
50515	'CHST11'	-0.951082588	0.02073292	1.33E-04
23531	'MMD'	-0.920192692	0.02073292	1.33E-04
10046	'MAMLD1'	-0.899185834	0.022379321	1.51E-04
10903	'MTMR11'	-0.89237316	0.026866287	2.06E-04
89927	'C16orf45'	-0.858208328	0.042422848	3.94E-04
9949	'AMMECR1'	-0.855438343	0.017886309	1.09E-04
360023	'ZBTB41'	-0.840555193	0.010124988	5.40E-05
1.01E+08	'NOTCH2NLB'	-0.828983403	0.026866287	1.98E-04
160897	'GPR180'	-0.82767099	0.006558552	2.90E-05
10783	'NEK6'	-0.811653125	0.005108144	2.06E-05
5865	'RAB3B'	-0.807377855	0.028251608	2.21E-04
85414	'SLC45A3'	-0.796951175	0.026866287	1.94E-04
79739	'TTLL7'	-0.792909854	0.010740117	5.85E-05
221656	'KDM1B'	-0.777258472	0.015250908	9.08E-05
6480	'ST6GAL1'	-0.762763565	0.047048275	4.61E-04
57613	'FAM234B'	-0.724012378	0.04736613	4.70E-04
84888	'SPPL2A'	-0.71382001	0.004264728	1.57E-05
22848	'AAK1'	-0.712743044	0.020393987	1.27E-04
22858	'ICK'	-0.708348343	0.026866287	2.04E-04
224	'ALDH3A2'	-0.706597313	0.0357682	3.00E-04
283431	'GAS2L3'	-0.701288474	0.035469913	2.96E-04
26579	'MYEOV'	-0.684551374	0.009090272	4.74E-05
214	'ALCAM'	-0.665687456	0.045276951	4.31E-04
284565	'NBPF15'	-0.66318288	0.04007252	3.49E-04
151230	'KLHL23'	-0.635181335	0.035781545	3.02E-04
5937	'RBMS1'	-0.621510984	0.021696188	1.41E-04
4780	'NFE2L2'	-0.612022025	0.003033304	9.80E-06

57590	'WDFY1'	-0.568784956	0.011303261	6.28E-05
7003	'TEAD1'	-0.553077176	0.041201418	3.78E-04
51361	'HOOK1'	-0.540224624	0.047048275	4.61E-04
835	'CASP2'	-0.517516	0.046687521	4.53E-04

Table S5. Proteomic mass spectrometry based on PRDX1 reactivity in HCT116$^{PRDX1-OE}$ *v.s.* HCT116^{CON-OE}

Protein	Name	No. of peptides
PLEC	Isoform 2 of Plectin	153
ACTG1	Actin, cytoplasmic 2	138
MYH11	Myosin heavy chain 11 smooth muscle isoform	74
MYO1C	Unconventional myosin-Ic	54
POTEF	POTE ankyrin domain family member F	37
MYO18A	Unconventional myosin-XVIIIa	35
POTEI	POTE ankyrin domain family member I	30
HSPA8	Heat shock cognate 71 kDa protein	27
POTEJ	POTE ankyrin domain family member J	27
SPTAN1	Spectrin alpha chain, non-erythrocytic 1	24
ACTN1	Alpha-actinin-1	22
BUB3	Mitotic checkpoint protein BUB3 (Fragment)	20
NUSAP1	Nucleolar and spindle-associated protein 1	20
MPRIP	Myosin phosphatase Rho-interacting protein (Fragment)	19
DBN1	Drebrin (Fragment)	19
MPRIP	Myosin phosphatase Rho-interacting protein	19
PLEC	Plectin (Fragment)	18
MLLT4	Afadin	17
KRT17	Keratin, type I cytoskeletal 17	17
TPM3	Tropomyosin alpha-3 chain	17
TUBB2B	Tubulin beta-2B chain	15
TUBB2A	Tubulin beta-2A chain	15
PRMT5	Protein arginine N-methyltransferase 5	15
SLC25A6	ADP/ATP translocase 3 (Fragment)	14
RBM33	RNA-binding protein 33 (Fragment)	14
SEPT2	Septin-2	14
SYNCRIP	Heterogeneous nuclear ribonucleoprotein Q	14
TPM2	Tropomyosin beta chain	13
EPB41L5	Band 4.1-like protein 5	13
TUBA3C	Tubulin alpha-3C/D chain	13
CCT2	T-complex protein 1 subunit beta	13
HSPA1L	Heat shock 70 kDa protein 1-like	13
PKM	Pyruvate kinase PKM	13

DHX35	Probable ATP-dependent RNA helicase DHX35	11
TPM2	Tropomyosin beta chain	11
SPECC1L	Cytospin-A (Fragment)	11
FARSA	Phenylalanine--tRNA ligase alpha subunit	11
TUBA3E	Tubulin alpha-3E chain	11
SPTY2D1	Protein SPT2 homolog	11
FLII	Protein flightless-1 homolog	11
TPM2	Tropomyosin beta chain	11
RPL7	60S ribosomal protein L7	10
AURKB	Aurora kinase B	10
ZC3HAV1	Zinc finger CCCH-type antiviral protein 1	10
TUBA3C	Tubulin alpha-3C/D chain	10
RDX	Radixin	10
TMPO	Thymopentin	9
UHRF1	E3 ubiquitin-protein ligase UHRF1	4
SMURF2	E3 ubiquitin-protein ligase SMURF2	4
ERBB2	Receptor tyrosine-protein kinase erbB-2	3
CUL2	Cullin-2	3
TCF7L2	Transcription factor 7-like 2	3
CAND1	Cullin-associated NEDD8-dissociated protein 1	3
UBAP2	Ubiquitin-associated protein 2	3
MID1	E3 ubiquitin-protein ligase Midline-1	3
UBP5	Ubiquitin carboxyl-terminal hydrolase 5	3
RNF20	E3 ubiquitin-protein ligase BRE1A	2
UBP36	Ubiquitin carboxyl-terminal hydrolase 36	2
KCMF1	E3 ubiquitin-protein ligase KCMF1	2
CUL1	Cullin-1	2
TXNRD1	Thioredoxin reductase 1	2
KAT7	Histone acetyltransferase	2
GSK3B	Glycogen synthase kinase-3 beta	2
